# Literary evidence for taro in the ancient Mediterranean: A chronology of names and uses in a multilingual world

**DOI:** 10.1371/journal.pone.0198333

**Published:** 2018-06-05

**Authors:** Ilaria Maria Grimaldi, Sureshkumar Muthukumaran, Giulia Tozzi, Antonino Nastasi, Nicole Boivin, Peter J. Matthews, Tinde van Andel

**Affiliations:** 1 Research Laboratory for Archaeology & the History of Art, University of Oxford, Oxford, United Kingdom; 2 Naturalis Biodiversity Center, Leiden, The Netherlands; 3 Faculty of History, Yale-NUS College, Singapore, Singapore; 4 Dipartimento dei Beni Culturali: archeologia, storia dell'arte, del cinema e della musica, Università degli Studi di Padova, Padova, Italy; 5 Dipartimento di Lettere, Arti e Scienze sociali, Università degli Studi “Gabriele d’Annunzio” of Chieti-Pescara, Chieti, Italy; 6 Max Planck Institute for the Science of Human History, Jena, Germany; 7 National Museum of Ethnology, Osaka, Japan; Missouri Botanical Garden, UNITED STATES

## Abstract

Taro, *Colocasia esculenta* (L.) Schott, is a vegetable and starchy root crop cultivated in Asia, Oceania, the Americas, Africa, and the Mediterranean. Very little is known about its early history in the Mediterranean, which previous authors have sought to trace through Classical (Greek and Latin) texts that record the name *colocasia* (including cognates) from the 3rd century BC onwards. In ancient literature, however, this name also refers to the sacred lotus, *Nelumbo nucifera* Gaertn. and its edible rhizome. Like taro, lotus is an alien introduction to the Mediterranean, and there has been considerable confusion regarding the true identity of plants referred to as *colocasia* in ancient literature. Another early name used to indicate taro was *arum*, a name already attested from the 4th century BC. Today, this name refers to *Arum*, an aroid genus native to West Asia, Europe, and the Mediterranean. Our aim is to explore historical references to taro in order to clarify when and through which routes this plant reached the Mediterranean. To investigate Greek and Latin texts, we performed a search using the *Thesaurus Linguae Graecae* (*TLG*) and the *Thesaurus Linguae Latinae* (*TLL*), plus commentaries and English and French translations of original texts. Results show that while in the early Greek and Latin literature the name *kolokasia* (Greek κολοκάσια) and its Latin equivalent *colocasia* refer to *Nelumbo nucifera* Gaertn., after the 4th century AD a poorly understood linguistic shift occurs, and *colocasia* becomes the name for taro. We also found that *aron* (Greek ἄρον) and its Latin equivalent *arum* are names used to indicate taro from the 3rd century BC and possibly earlier.

## Introduction

Taro, *Colocasia esculenta* (L.) Schott,(Fam. Araceae) has a likely natural range extending from Southeast Asia to Australia and Papua New Guinea [1–4] and is now distributed as a cultivated vegetable and root crop (producing corms) in tropical to temperate regions of the world [2–3,5]. While the geographical origins of cultivated taro within its natural range remain uncertain, the Asian origin of taro as a species is clear [3, 4]. Its introduction to the Americas is historically modern, and was primarily from Africa via the slave trade, while its presence in Africa is ancient with Egypt (and thus the eastern Mediterranean) considered a possible route of introduction [6]. The plant is primarily grown for its edible, starchy corms, but in many areas the leaf blades and petioles are also eaten (in all cases, with cooking to remove an acrid, ‘itchy’ factor).

Within the Mediterranean, including southern Europe, taro is widely grown but its use as a food crop is now largely confined to the eastern Mediterranean (southern Turkey, Cyprus, the Levant, and Egypt). Taro was previously grown as a food plant in Italy, Portugal, and Spain [7], but it is now naturalized and mainly used as an ornamental plant, embellishing fountains and ponds. The origins of taro cultivation in the Mediterranean remain unclear, despite the existence of many written records [2]. The single archaeological finding of taro in this region consists of fragments of corm tissue found in Egypt and dating from ca. 1000 AD [8–9], long after the earliest written indications of taro. The exceptionally long historical and linguistic record relating to taro in the Mediterranean has never been comprehensively investigated, and is reviewed here with the aim of understanding spatial and temporal aspects of the introduction and spread of taro in the Mediterranean basin. We use modern historical sources to introduce the current geographical distribution of taro in the Mediterranean, and then consider (possible) attestations of taro in early Greek, Latin, Hebrew, and Arabic texts. Like other writers of the era, Renaissance botanists were keenly interested in classical (Greek and Roman) sources, for information on plants, but also began to re-interpret, supplement and expand the earlier writings with their own field observations and plant illustrations [10]. The Linnaean genus name for taro, *Colocasia*, has ancient roots as a Greek vernacular name, but early usage of the name has been a matter of debate for centuries [11–12], in part because of its connection with another plant of deep historical interest [13], the sacred or Indian lotus, *Nelumbo nucifera* Gaertn.

The scientific naming of taro has been reviewed by Hill [14], Plucknett [15], Hay [16], and Orchard [17]. Modern confusion in the naming of species within the genus *Colocasia* resulted in part from the fact that Linnaeus and subsequent authors based many of their descriptions and names on cultivated forms produced by human selection. Here we focus on the genus name *Colocasia*, which is derived from the Greek vernacular name *kolokasia* (κολοκάσια). Through a prolonged debate on the origins and original meanings of this Greek name [12–13, 18–25], a change in the usage of *kolokasia* from an earlier name for the edible root of *N*. *nucifera* in Egypt to a later name for taro, has been recognized by most scholars. Botanists in the 16th and 17th centuries generally followed Classical sources in identifying taro as a kind of *arum* and describing the plant under names such as *Egyptian arum* or *edible arum*. Linnaeus [26] distinguished two kinds of taro with the names *Arum colocasia* and *Arum esculentum*, and these were later combined under the genus name *Colocasia* by Schott [27]. Our analysis of early sources that predate modern European botany has clarified when taro reached the eastern Mediterranean, and raises the possibility of multiple early introductions.

## Methods

Many early historical references to taro are now available online. We referred mainly to the *Thesaurus Linguae Graecae* [28], a digital corpus of Greek texts from Homer (8th century BC) to the fall of Byzantium (1453 AD), and the *Thesaurus Linguae Latinae* [29], a Latin lexicon which includes all Latin texts from the classical age to the 7th century AD. We used the following names in our query: a) the Greek *kolokasion* (κολοκάσιον, plur. κολοκάσια) and the Latin equivalents *colocāsium* or *colocāsia*; b) the Greek term for *Egyptian bean* (κύαμος Αἰγύπτιος) and its Latin equivalent *cyamos Aegyptius*; c) the Greek *ouingon* (οὔϊγγον), with minor orthographic variations (οὔϊπον, οὔϊτον), and the Latin form *oetum*, and d) the Greek *aron* (ἄρον) and the Latin equivalents *aros*, *arum* and *aron*.

Additionally, we have used other textual sources from late medieval to recent times, especially the works of herbalists and botanists. Of particular importance are Matteo Silvatico [30], Gaspard Bauhin [31], Pietro Mattioli [32] and Carolus Linnaeus [26].

The early sources that we consulted predate the Linnaean codification of floral and faunal taxonomies in the mid-18th century, and the spread of an international standard system of botanical nomenclature. Early names and descriptions of plants, and particularly those far removed in time, do not necessarily correspond to their modern equivalents. Vernacular and scholarly uses of plant names change over time, and vary among individuals and in different communities. To establish congruence between the early and modern meanings of names, we must interpret a range of linguistic and contextual evidence. In the present paper, the name “taro” will only be used when an earlier name is thought to definitely refer to *Colocasia esculenta* (L.) Schott. We based the modern distribution of taro in the Mediterranean on the records of *C*. *esculenta* in botanical treatises, herbarium collections, ethnographic and agricultural reports from 1854 to the present [S1 Appendix]. Information on modern distribution, variation and uses was also gathered through original fieldwork in the region (Matthews 1996–2001, Grimaldi 2010–2017). The early sources investigated in this text are cited according to the standard referencing code adopted in Classical Studies. In Table 1, we report in chronological order the texts studied followed by the abbreviations used in this paper, the terms examined and their interpretations. Translations and commentaries of each text are also listed.

**Table 1:** Textual sources for *arum*/*colocasia*.

Author	Date	Title	Term(s) investigated and interpretation	Reference/ Translation
**Herodotus**	5th c. BC	*Histories* (*Historiae*)	‘*lotos*’ (*Nymphaea lotos* L.), (*N*. *cerulean* Sav.) lily with a rose-like flower and the fruit resembling a wasps’ comb (*Nelumbo nucifera*) ‘*byblus*’ (*Cyperus papyrus* L.)	[33]
**Corpus Hippocraticum**	5th-4th c. BC	*On Ulcers* (*De Ulceribus = Ulc*.) *On Diseases* (*De Morbis = Morb*.)	‘*big aron*’ (possibly taro)	[34]
**Aristotle**	4th c. BC	*Inquiries on Animals* (*Historia Animalium* = *HA*)	‘*aron*’ (wild aroid, eaten by bears)	[35]
**Theophrastus**	4th-3rd c. BC	*Enquiry into Plants* (*Historia Plantarum = HP*)	‘*edible aron*’ (possibly taro)	[36]
**Diphilos of Siphnos**	3rd c. BC	*On Diet fit for Persons in good and bad Health* (*De rebus aegrotantibus et bene valentibus sumendis*)	‘*kolokasion*’ (root of *Egyptian bean*, i.e. lotus)	[37]
**Nicander of Colophon**	2nd c. BC	*Georgics* (*Georgica*)	‘*kolokasion*’ (root of *Egyptian bean*, i.e. lotus)	[37]
**Virgil**	1st c. BC	*Eclogues* (*Eclogae* = *Ecl*.)	‘*colocasia*’, poetic (ambiguous, but likely lotus)	[38]
**Strabo**	1st c. BC-1st c. AD	*Geography* (*Geographia* = *Geogr*.)	‘*korsion*’ root of water lilies	[39]
**Columella**	1st c. AD	*On Agriculture* (*De Re Rustica*)	‘*colocasia*’, planted as ornamental in pond (ambiguous)	[13, 40]
**Pliny the Elder**	1st c. AD	*The Natural History* (*Naturalis Historia* = *NH*)	*‘colocasia’* (an alternative name for *Egyptian bean*, i.e. lotus);*‘arum of Egypt’* (two kinds; one possibly taro)	[41–43]
**Dioscorides Pedanius**	1st c. AD	*Medical Materials* (*Materia Medica* = *MM*)	*‘kolokasion’* (root of *Egyptian bean*, i.e. lotus)*; ‘aron*’, also known as loufa’(often interpreted as taro, but description does not match)	[32, 44–46]
**Martial**	1st-2nd c. AD	*Epigrams* (*Epigrammata*)	‘*colocasia*’, edible part fibrous (as in lotus)	[47–48]
**Galen of Pergamon**	2nd c. AD	*On the Properties of Foodstuffs* (*De Alimentorum Facultatibus* = *Al*. *Fac*.)	‘*kolokasia*’ (lotus root); ‘*aron*’ (two kinds, ‘*Cyrenaic aron*’ apparently taro)	[49]
**Athenaeus of Naucratis**	2nd-3rd c. AD	*The Banquet of the Learned* (*Deipnosophistae*)	‘*kolokasion*’ (lotus root)	[37]
**Judah the Prince**	2nd c. AD	*Mishnah*	‘*qarqas*’ or ‘*qeriqas*’ (Hebrew: קַרְקָס) (conventionally interpreted as taro)	[50]
**Apicius**	2nd-4rd c. AD	*On the Subject of Cooking* (*De Re Coquinaria*)	‘*colocasia*’ (likely taro)	[51]
**Palladius**	4th c. AD	*Agricolture* (*Opus Agriculturae = Op*. *Agr*.)	‘*colocasia*’ (likely taro)	[52]
**Jerusalem Talmud**	4th-5th c. AD	*Jerusalem Talmud*	‘*kolkasyah*’ *or* ‘*kolkas*’ (Hebrew) (taro)	[53]
**Aetius of Amida**	6th c. AD	*Sixteen Books on Medicine* (*Libri medicinales*)	‘*kolokasion*’, medicinal root (likely taro)	[28]
**Paul of Aegina**	7th c. AD	*Medical Compendium* (*De Re Medica*)	‘*culcas*’ (likely taro)	[30]
**Mesue the Elder**	8th-9th c. AD	*Book of Simples* (*Liber de Simplicibus*)	‘*qolqas*’ (taro)	[30]
**Isaac Israeli**	9th-10th c. AD	*On Particular Diets* (*Diaetae Particulares*)	‘*qolqas*’ (taro)	[30]
**Ibn Sīnā (Avicenna)**	10th-11th c. AD	*The Canon of Medicine* (*Liber canonis medicinae*)	‘*qolqas*’ (taro)	[30]
**Ibn Wāfid (Serapion)**	12th c. AD	*The Book of Simple Medicaments* (*Liber aggregatus in medicinis simplicibus*)	‘*hulcas*’, ‘*chulcassia*’ (taro)	[30, 54]
**Matteo Silvatico**	1317 [1474]	*Opus Pandectarum Medicinae or Pandectae Medicinae*	‘*culcasia*’, ‘*culcas*’, ‘*collocasia*’, ‘*hulcas*’, and ‘*caso*’, (taro)	[30]
**Nicolò Roccabonella**	1445–48	*Liber de Simplicibus*	‘*Faba Aegyptia*’, ‘*Faba Syra*’, ‘*Culcasia*’ (taro)	[55]
**Luigi Anguillara**	1561	*Semplici dell’eccellente*	‘*Colocasia*’ (taro)	[56]
**Pietro A. Mattioli**	1565, 1580	*Commentarii*	‘*Arum Aegyptium*’ (taro)	[32]
**Andrea Cesalpino**	1583	*On Plants* (*De Plantis*)	‘*aron magnum*’ (big *arum*) (taro)	[57]
**Leonard Rauwolf**	1583[1693]	*A collection of curious travels*. * *.* *.*in the Eastern countries*.	‘*Egyptian bean*’ ‘*colocasia’* (taro)	[58–59]
**Prospero Alpini**	1592	*De Plantis Aegypti liber; De Plantis Exoticis*	‘*culcasia*’ (taro)	[60]
**Paolo Boccone**	1674	*Observations Naturelles*	‘*Arum Aegyptium*’, ‘*Culcasi*’ (taro)	[61]
**Carl Linnaeus**	1753	*Species Plantarum*	‘*Arum Colocasia*’ ‘*Arum esculentum*’ (taro)	[26]

Chronological sequence of the Classical, Byzantine, Arabic, Medieval and Renaissance authors, who mentioned *kolokasia* (Greek κολοκάσια), *colocasia*, *aron* (Greek ἄρον) and *arum* in the Mediterranean. Titles of sources investigated are given in English and in Latin with their abbreviation, if present. Usage, interpretation or possible interpretation is given in brackets.

## Results

### Modern naming, distribution and uses

**Fig 1:**
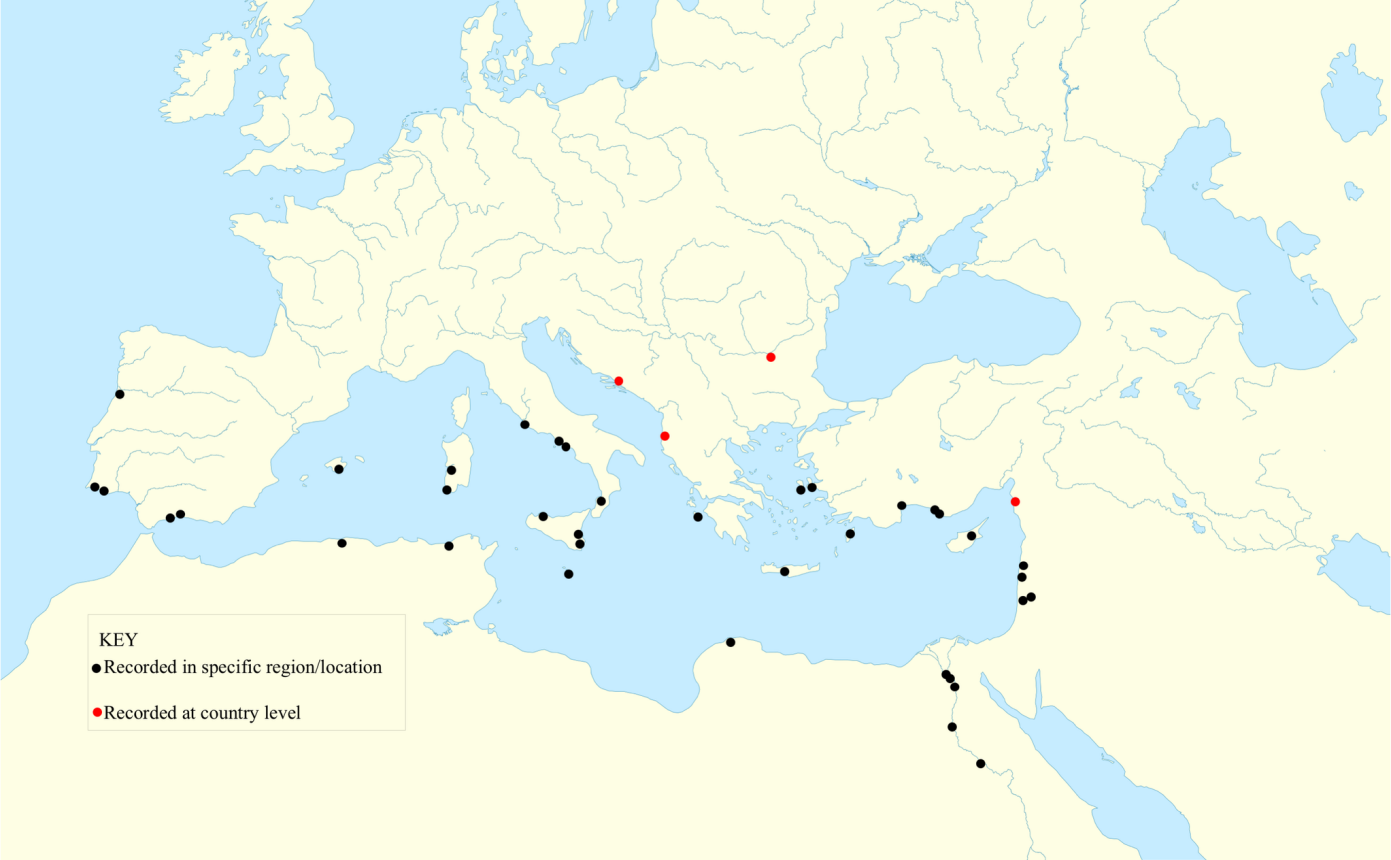
Taro Mediterranean map. Map of taro distribution in the Mediterranean, based on sources reported in the Appendix 1 [S1 Appendix].

Today, taro is widely distributed across the Mediterranean (Fig 1). It is extensively cultivated in Egypt [2, 62] where it is a common root crop known by the Arabic name *qolqas* (القلقاس) and in Cyprus, where it is known by the Greek name *kolokasi* (κολοκάσι) [2]. These two names and their cognate forms are predominant in the eastern Mediterranean and the Levant. Our recent field studies suggest that a single morphological form of taro is currently widespread in the eastern Mediterranean, from Egypt to Cyprus, Greece, and Italy. This plant fits an earlier description of a common, standard cultivar grown in Egypt, locally known as *baladay* or *masry* [63], in contrast to a cultivar introduced in the early 20th century known as *qolqas americani* [64]. We suspect that the common cultivar is a single, widespread clone and an ancient introduction, but further fieldwork and genetic analysis are needed to confirm this. In Europe, other morphotypes of taro are present in botanical gardens and city markets, with corms imported from Africa, Asia, and the Americas. A naturalized purple-stemmed form of unknown origin has also been reported in Spain [65]. Diverse immigrant communities across the Mediterranean and Europe import taro from their home regions as a traditional food, so new varieties may appear on a regular basis.

### Dating an early semantic shift from sacred lotus to taro

In early classical literature, descriptions of the aquatic waterlilies (*Nymphaea* spp.) and sacred lotus (*Nelumbo nucifera*) are clear, but clear descriptions of the semi-aquatic taro are lacking. Names for the former refer to the whole plant or a distinctive part: *lotos* for *Nymphaea lotus* (white waterlily) and *N*. *caerulea* (blue waterlily), and *kyamos* (bean) and *Egyptian bean*, for *Nelumbo nucifera* [13, 18–19, 23–24]. The large bean-like seeds of sacred lotus are produced in a fruiting head called *kiborion* (κιβώριον). Carbonized fruits of *N*. *nucifera* have been recovered from a late 4th century BC necropolis at Salamis in Cyprus [66]. Desiccated seeds of sacred lotus have also been found at Berenike (Egypt) and dated to the 1st-2nd century AD [67]. The two waterlily species are considered to be native of the Nile delta, while sacred lotus is generally considered as an ancient introduction from India [13]. The natural range of sacred lotus is believed to extend from India to East Asia [68].

Herodotus [33] in his *Histories* gave a detailed account of the life of people living in marshes of the Egyptian delta. He described the uses of wetland plants ‘with poppy-like flowers and edible roots’ (*Nymphaea* sp.), *Nelumbo nucifera* ‘with fruit in a calyx that looks like a comb made by wasps’, and *byblus* (*Cyperus papyrus* L.), of which the lower part could be roasted and eaten, while the upper stem was put to other uses (*Hist*. 2,92). Theophrastus also wrote on Egyptian plants and noted that the lotus root (*N*. *nucifera*) was a staple food of people living in wetlands who planted the seeds to establish perennial lotus beds in swamps and lakes (*HP* 4,8,8) [36]. Theophrastus used the term *korsion* (κόρσιον, (etymology unknown, but possibly “small root”) to identify the root of a waterlily (*Nymphaea* sp.). The same term was later used by Strabo to identify the root of Egyptian lotus (*Nymphaea* sp.), after a discussion of the edible foods peculiar to Egypt (*Geogr*. 17,2) [39]. Although taro may have been present in Egypt by this time, its absence in the otherwise detailed descriptions of wetland food plants suggests that it was not common.

Two later writers of the 1st century AD, Dioscorides and Pliny the Elder, used the name *kolokasia* (or *kolokasion*) to identify the edible starchy corms or rhizomes (‘roots’) of *Nelumbo nucifera*. They and other writers in the early centuries AD do not use this name in relation to taro or other plants, and there is no reason to doubt their application of the name [S1–S7 Texts]. Woenig [19], in a detailed botanical and historical account of *N*. *nucifera* in Egypt, explained the ease with which the plant must have naturalized after its introduction as a useful food plant at around the time of Herodotus. The use of *Nymphaea* spp. as food plants continued in the Nile delta to the 19th century, but the sacred lotus was no longer present, and may have disappeared hundreds of years earlier [18–19].

As a name for Indian sacred lotus, *kolokasia* (κολοκάσια) seems to have fallen out of use in Late Antiquity sometime after the 2nd century AD, and possibly around the 4th-5th centuries AD, when a poorly understood semantic shift took place in extant literary uses of the name. From this period onwards, the name *kolokasia* was applied to taro instead. In the Eastern Mediterranean today, *kolokasia* and its cognates in modern Greek (*kolokasi*), Latin (*colocasia*), Turkish (*gölevez*, *kolokas*, *gologas*) and Arabic (*kulkas*, *qolqas*) are widely used but refer only to taro. The semantic shift in the meaning of *colocasia* appears to follow the first records of other names that are considered definite references to taro. To investigate this semantic shift, we look into the Near East region, in particular to Hebrew texts that mention taro and then examine Greek and Latin sources in more detail.

### Hebrew literature: The Mishnah and the Jerusalem Talmud

**Fig 2:**
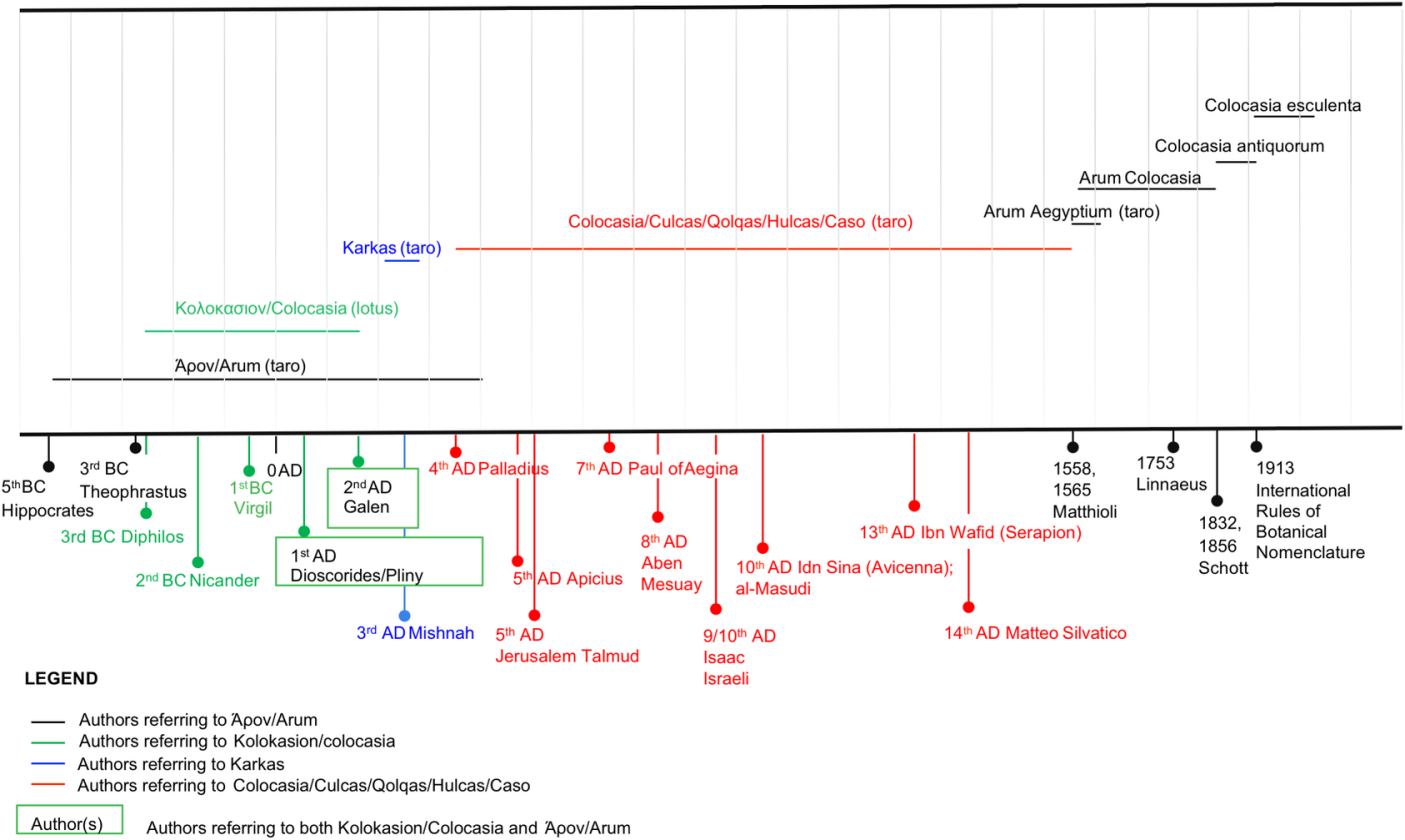
Taro timeline. Timeline for the use of the names *Arum* and *Colocasia* from Herodotus (5th century BC) to the International Rules of Botanical Nomenclature (1913).

The crop named *qarqas* or *qeriqas* in the *Mishnah* [50], the earliest work of rabbinical literature, has conventionally been interpreted as taro, at least from the time of Maimonides (1135–1204), based on later equations of *qarqas* with *qolqas*, the Arabo-Hebraic term for taro (Ma'as. 5:8: הקרקס אף אומר מאיר ר) [50] (Fig 2). Taro is known as *korkasi* in Coptic [64], which is is probably a borrowing from the Hebrew *qarqas* or the Arabic variant *qorqas*. A later reference to taro in the Near East appears in the *Jerusalem Talmud* (TJ), in which Jewish rabbis discussed the liability of *qolqas* (taro) to tithing, thus suggesting that it was known as a cultivated crop (TJ Nedarim 7:1, 40b) [53]:

“Rabbi Isaac ben Haqolah and Rabbi Joshua ben Levi both say that taro [*qolqas*] is like a vegetable for tithes”.

The Hebrew *qolqasyah or qolqas* are first attested in the rabbinical literature (TJ Shev. 2:10, 34b and TJ Pe'ah 1:5, 16c, respectively, [53]) and are undoubtedly cognate with the earlier Greek *kolokasia* and the later Arabic *qulqas* or *qolqas*. Traditionally, the Hebrew *qolqasyah or qolqas* are interpreted as taro [50], suggesting that the semantic shift from lotus to taro took place in the Eastern Mediterranean when the lotus became less familiar in Late Antiquity [cfr. S8 Text].

### From garden to table: Interpreting *colocasia* in late antiquity

In late Roman texts, the meaning of *colocasia* becomes ambiguous, or more likely to refer to taro. In his agricultural treatise Palladius mentions *colocasia* first as a plant from which ‘bulbs’ are set in the month of February (*Op*. *Agr*. 3,24,14) [52]:

“We are to set the bulbs of the colocasia this month: it likes a moist rich situation well watered. It grows luxuriant round fountains and rivers, and does not care about the quality of the soil if it is cherished by perpetual moisture. It may be kept almost always in a flourishing condition if it is defended from the cold, as the citron-plantations are protected, by shelter.”

In a second mention by Palladius, *colocasia* is simply listed in a section on plants to be planted in gardens in March (*Op*. *Agr*. 4,9,5), early spring in Italy. André [69] argued that Palladius’ *colocasia* is taro. The planting conditions described by Palladius appear to match those that are optimal for taro, which is often grown in soil to which water is added, or next to water, in contrast to lotus, which is typically grown in standing water. Under temperate conditions, lotus typically enters full dormancy, without leaves, whereas taro can easily be maintained in leaf, or ‘flourishing condition’, if it is kept warm.

Around the same time, recipes associated with the name of Apicius are collected in a work entitled *On the Subject of Cooking* that includes three references to *colocasia* (3,68; 6,218; 7,325) [51]. Although attributed to Apicius, the work is probably a collection of recipes emanating from several persons between the 2nd and 4th centuries AD. The word *colocasia* is attested in several variant forms, probably representing orthographic mistakes in Apician manuscripts, including *coladium*, *coledium*, *coloesium* and *colesium* [51]. The compendium includes advice on how to use the corms of *colocasia* to bulk out meat or fowl dishes, suggesting it was not a major element in the diet of ordinary Romans. According to Gentilini [70] and Vehling [51] this is taro, and the recipes can indeed easily be applied to taro and have parallels with later Middle Eastern, particularly Egyptian, recipes recorded in medieval Arabic [71]. There is also an echo here of a recipe given by Galen for an edible *arum* (see below).

### The unusual aubergine of Aetius of Amida

Aetius of Amida, a court physician writing in Greek (527–565 AD), described *kolokasia* in a manner that suggests taro rather than lotus (*Med*. 1,210) [28]; translation by IMG:

“Kolokasion or aubergine. The strength of the root is similar to that of the turnip and the onion, its body is sticky so that it is used for cleansing and for easing the bowels.”

The mention of aubergine or eggplant (*Solanum melongena* L.) is difficult to interpret here, as the plant has no obvious external resemblance to taro, *Nelumbo* or *Nymphaea*. Perhaps the plant shares physical or medicinal properties with the root of *kolokasion*. The description of a root that is ‘sticky’ and a remedy for gastrointestinal diseases is consistent with taro, which is described as having such utility by Arabic authors. Aetius also lists *kolokasia* alongside *aron* and *dracontium* (both aroids) in a list of plants with aphrodisiac properties (*Med*. 11,35), suggesting that he used *kolokasia/kolokasion* to identify an aroid (i.e. taro) and not the rhizome of the sacred Indian lotus.

### The medicinal culcas of Paulus

A reference to a plant known as *culcas* is also found in a passage attributed to Paulus of Aegina, one of the last Greek physicians of the Late Antique period [30].

“*Culcasia* is a plant known by everyone. It springs up near the water; its root once cooked and eaten is very useful to the stomach”.

Paulus was born in Greece on the island of Aegina in 625 AD and practiced in Alexandria [72]. He was cited by Matteo Silvatico, a medieval doctor, who wrote the *Opus Pandectarum Medicinae*, also known as *Pandette* [30], a text written in 1317 but published only in 1474 as an encyclopedic work documenting the knowledge of plants at the time [S9 Text]. The passage by Paulus, cited by Silvatico, is not found in the *Seven Books on Medicine*, which is the only work of Paulus that has survived. In this book, there is only a reference to the *Egyptian bean* (Greek κύαμος Αἰγύπτιος), which does not correspond to Silvatico’s description of *culcasia*. Part of Paulus’ text or other texts by him may be missing, as it seems unlikely that Silvatico would have inserted a text and inaccurately attributed it to Paulus. Indeed, later Syriac and Arab authors who took great interest in his work reported that Paulus of Aegina had also written *The Therapy of Children*, of which most was lost except for parts of text that survived in a number of Arabic fragments [73].

### A thousand and one qolqas in medieval Arabic literature (8th-13th century)

The reign of the early Abbasid caliphs, beginning in the 8th century, saw many exchanges of Greek and Arab scientific knowledge. Pioneering Arabic works on botany and pharmacology were much indebted to Greek authors such as Galen and Dioscorides. In these and later Arabic texts, taro is clearly attested as *qolqas* (and its cognates). Silvatico [30], Täckholm and Drar [64], Portères [24], and Lev [74], refer to numerous Arabic authors of the medieval period who regard *qolqas* as a food and medicinal plant. Silvatico [30] mentioned the Arabic physicians Yahanna ibn Masawaih (8th century), Israel Isaac (9th-10th century), Ibn Wāfid (13th century), and the philosopher and writer Ibn-Sīnā (10th-11th century). Lev [74] cites the geographers al-Muqaddasi (10th century), al-Dimashqi (1256–1327), al-Badri (15th century), the Egyptian mathematician al-Qalqashandi (1355–1418), and the Syrian physician Daud al-Antaki (16th century) as authorities on taro. The ‘A thousand and one qolqas’ in our sub-heading is an allusion to the many references to taro in the Arabic literature, but also to the actual presence of *colocasia* (*kulkasá*) in the *Arabian Nights*: ‘I also bought colocasia roots, fried and soaked in honey’ [75]. Lewicka [71], notes that fried colocasia corms were a favorite in Medieval Cairo. This passage in the *Arabian Nights* probably comes from a similar chronological/spatial horizon (late 13th-14th century Mamluck Egypt/Syria), where fried taro chips could have been the medieval equivalent of French fries.

Taro was certainly present in Egypt from ca. 1000 AD onwards. The desiccated remains of taro were recently discovered at the archaeological site and ancient port of Quseir al-Qadim and gave calibrated radiocarbon dates between 1050–1170 AD [8–9]. However, the absence of taro in the Geniza documents suggests that it was not traded as a commodity through the port [9]. Instead, overland traders may have brought it from elsewhere. In the 9th century AD, Abū Hanīfa al-Dīnawarī, the Iranian author of the *Book of Plants* [76] refers to *qolqas* as both a food and a medicinal plant. Al-Masʿūdī’s 10th century account [77] during a journey along the coast of eastern Africa is also informative:

“The Zanj eat bananas, which are abundant there and in India, but the base of their nutrition is the *dorrah* and *kalari*, which they pull from the earth like a truffle and like the root of horse-heal. It is widely found in Aden and in the region of Yemen nearby this town; it resembles the Egyptian and Syrian *colocasia*”.

Zanj is the name used by medieval Arabic geographers to refer to Bantu-speaking people living along the Swahili coast of eastern Africa, and *kalari* is presumably cognate with the Malay name for taro, *keladi* [7]. The comparison with horse-heal (*Inula helenium* L) may derive not only from the similarity of harvesting method (pulled from the earth as a root crop), but also because horse-heal was propagated vegetatively, had bitter or acrid properties, and was used as both food and medicine (see Pliny, *NH* 19, 91) [41].

Abū ‘l-Khayr al-Ishbīlī, an 11th-century agronomist born in Seville, mentioned the use of taro (*qulqās*, *qulqāṣ*) as an ornamental plant [78]. In the 12th century, the Sevillian Ibn al-‘Awwam stated in a chapter on root vegetables that taro (*qorqas*) grows in stagnant and brackish waters rich in nutrients, does not produce flowers or fruits, and has two types: one producing a round and the other an elongated root. These shapes may refer to the round mother-corms and elongate side-corms produced by many taro cultivars today. The root was dug out ‘like a turnip”, chopped into pieces, and cooked with meat [79]. Also in the 12th century, the Egyptian historian Al- Maqrīzī wrote that taro was planted with sugar cane, and, on the authority of an earlier author, Alī ibn Riḍwān (c. 988-c. 1061 AD), noted that taro was cultivated in the Nile Delta [80]. ʿAbd al-Laṭīf al-Baghdādī, an early 13th-century author, reports first-hand of seeing taro in Egypt and Syria (Damascus). He also notes that it grew in Yemen and described in detail the corm, leaf, habit, dimensions, and its preparation, including its acridity and the need for peeling and cooking to render the corms edible, and the use of raw corms as medicine [81–82]. ʿAbd al-Laṭīf al-Baghdādī also saw that when left to dry, the taro corm becomes dry and woody. The archaeological specimens of taro recently found in Egypt were preserved in this woody state [9].

### Minerva’s garden and the book of simples

Not mentioned in previous discussions of the history of taro in the Mediterranean, are the work of the medieval author Matteo Silvatico, and the garden known as Giardino della Minerva (Minerva’s Garden) in Salerno (Italy). Silvatico was a physician whose interest in plants led him to build a terraced garden in the 14th century around the medieval walls of Salerno. This early botanical garden has been maintained as a living collection until today, despite changes in ownership and function. In Italian, this type of garden is also referred to as *Giardino dei semplici* (Garden of simples), where simple is a medicine extracted from one herb as opposed to a compound of herbal extracts mixed by an apothecary or doctor. Cultivating his plants and experimenting with their properties, Silvatico bundled his knowledge in his *Pandette* [30], regarded as an encyclopedic work documenting the knowledge of plants known at the time. Silvatico mentioned previous scholars’ knowledge about *colocasia* in chapter 197, where he introduced the synonyms *culcasia*, *culcas*, *collocasia* (Greek), *hulcas* (Arabic), and *caso* (Latin) for a plant that was present in his garden [S9 Text]. This text provides an invaluable link to descriptions of *colocasia* in the Classical period, the Islamic world and the early Renaissance in Italy. Silvatico’s work does not pretend to be a commentary, but more of a collection of all known information about plants, regardless of whether previous authors referred to the sacred Indian lotus or taro. Silvatico, who knew the references to the *Egyptian bean* (*N*. *nucifera*) in the likes of Pliny and Dioscorides, must have noticed the discrepancy between early Classical usages of this term and later medieval accounts.

In Silvatico’s text, *hulcas* was said to be well-known in Egypt among merchants who used to travel to Syria, which suggest that the plant originally reached Egypt from that country. This information may come from the 11th century *Book on Simple Drugs* [30, 54, 83] of Ibn Wāfid, a physician and pharmacologist in Toledo who collected and translated the medicinal texts of Dioscorides and Galen. An illuminated copy was made by Niccolò Roccabonella between 1445 and 1448 and offered as a botanical text to his son who was about to become a doctor [84]. It contains an extraordinary collection of plant drawings made by the painter Andrea Amadio, each accompanied by a brief description compiled by Roccabonella. After examining many of the oldest illuminated European herbals we conclude that this book contains the oldest surviving image of taro [55]. The plant has no inflorescence but shows large green leaves and a large underground corm, and despite inaccuracy in the leaf shape, the drawing overall displays other traits of taro very clearly. In this text, Roccabonella stresses the medicinal use of this plant more than its use as food, and it gives interesting hints on harvesting periods and the way taro was stored during that time [S10 Text].

Silvatico’s and Roccabonella’s accounts are invaluable historical resources, revealing an extensive knowledge of taro during the Middle Ages. Their works provide precious hints on the uses and terminology applied to taro in Latin, Greek and Arabic. Only the name *Syrian bean* (*Faba Sira*) is mentioned for the first time. The plant described by Silvatico in the *Pandette* still grows in the Garden of Minerva today and occupies a central position there [85].

### The *ouingon* of Theophrastus

Some authors have argued that the term *ouïngon*, used by Theophrastus in his *Historia Plantarum* refers to *C*. *esculenta*, and becomes *oetum* in Pliny (*NH* 21,88) [42]. This interpretation comes from inaccurate quotations of Theophrastus’s text [86], and has been assimilated to the point that in the Greek Lexicon *ouïngon* is defined as *Colocasia antiquorum*, a synonym of *C*. *esculenta* [87]. Theophrastus [36] referred to *ouïngon* as a plant known in Egypt and bearing underground fruits (*HP* 1,1,7) that are not regarded as roots (*HP* 1,6,9), and that possess large leaves and long edible tubers that men gather from the Nile when the river retreats (*HP* 1,6,11). While Amigues [88] accepted this as a description of taro, Täckholm and Drar [64] considered it to be an unidentified plant with different characteristics. Pliny’s *oetum* is a plant with a big root, but with only a few small leaves (*NH* 21,88) [42], which is enough to rule out taro, a plant with numerous large and broad leaves. Theophrastus’ description of the plant could refer to taro, or perhaps some other tuberous plant in the Nile valley. At present, the *ouingon* of Theophrastus remains a semantic conundrum.

### Classical *arum* and *aron*

The name *arum* is still commonly used in Europe for a genus related to taro and may have been used for taro at an early date. In classical texts, taro may have been known as *aron* or *arum* [56–57, 64, 89]. The Latin *arum* is an old name from which the family Araceae and the genus *Arum* derive their names. In the ancient world, *arum* was not a species-specific term; it referred to several morphologically similar plants with acrid corms and leaves with medicinal properties. All aroids by definition belong to the *arum* family, so it is inherently difficult to distinguish references to taro from references to other aroids when there is no accompanying description or image with sufficient detail to identify a species or genus. Here we focus on texts that suggest that taro may have been known in the eastern Mediterranean as a kind of *arum*, before the spread of other names for the plant (Table 1).

#### The big *arum* of Hippocrates

The *Corpus Hippocraticum* [34] is a collection of medical works written in the style of Hippocrates, and believed to date from Classical antiquity (5th-4th century BC) (Fig 2). Here, *arum* is a medicinal plant used to cure severe inflammation of the lungs (*Morb*. 2,47; 3,15–16), and to soothe burns (*Ulc*. 12,16,22). In one case (*Morb*. 2,47), the root of the *arum* “large as a vertebra” is recommended, indicating a rather small root, different from the large swollen root of cultivated taro. In other cases, the root of a “big *arum*” is suggested (*Morb*. 3,15–16). The different sizes might reflect various growth stages of one plant species or the existence of two different kinds of *arum*. Early scholars [90–91] interpreted *arum* as a wild species such as *Arum maculatum* L. or *A*. *italicum* L., while Littré [92–93] considered it to be *Arum Colocasia* (an old botanical name for taro). More recently, *A*. *maculatum*, *A*. *italicum*, and *C*. *esculenta* have all been proposed as candidates for the *arum* discussed in the *Corpus* [94]. In modern Turkey, fresh leaves or dried parts of *A*. *italicum* are still used as food, while its tubers and dried fruits are used to treat rheumatism and hemorrhoids [95].

#### Aristotle and Theophrastus: Wild and edible *arum* in the Peripatetic school

Aristotle, in his *Inquiries on Animals* [35], referred to *aron* as a root eaten by bears after hibernation to open up their constricted stomach after having little food for a long period (*HA* 600b11, 611b). This suggests a plant that was part of the natural flora. A different wild aroid, skunk cabbage (*Lysichiton* sp.), is known as part of the diet of bears in North America in early spring. Theophrastus [36] described the root of the *arum* as fleshy (*HP* 1,6,7), stout and fibrous (*HP* 1,6,8), smooth, loose and soft throughout and without bark (*HP* 7,9,4). However, he also mentioned an *edible arum* with big leaves (*HP* 7,13,1–2) and notes that once the leaves and the roots are boiled in vinegar, they become sweet and are good for fractures (*HP* 7,12,2). This *edible arum* had no stem or flower (*HP* 7,13,2), so it might refer to taro under cultivation, since cultivated taro is generally harvested before inflorescences develop [2]. A recent review of the genus *Arum* revealed that the eastern Mediterranean and the Balkans to the Near East form the center of greatest diversity for the genus [96], with a total of 28 species [97]. None of the *Arum* species are said to be flowerless, and this is true for all wild aroids. Taro is, therefore, a good candidate for Theophrastus’s *edible arum*.

#### Did Dioscorides ever see taro?

Dioscorides described the properties of *arum* in *De Materia Medica* (*MM* 2,197) [41]:

“The arum, which the Syrians call loufa. It sends out leaves similar those of the dragon arum, but smaller and without spots, a stem one span tall, purplish and pestle-shaped, upon which the saffron-colored fruit grows; the root is white tending toward the root of dragon arum; it too, is eaten boiled, although it is less pungent. Its leaves are cured for eating; they are also eaten boiled after they have dried by themselves.

The seeds, leaves, and root have the same properties as dragon arum. Particularly the root, applied with bullock's dung to those troubled with gout, does them good. The root, plastered on, is efficacious for the gouty. It is stored the same way as the root of dragon arum and, in general, it is edible because it is not very pungent.”

The description of fruit color, and height of the fruiting stem (one span, ca. 20 cm) match the traits of *Arum maculatum*, which is widespread in Europe and West Asia and known as a source of edible starch after acridity has been removed [98]. Nevertheless, the arum ‘which the Syrians called loufa’ is almost certainly the Solomon’s lily (*Arum palaestinum* Boiss.). The same name (*luf*) is regularly cited in rabbinical texts [50], which indicate that both the leaves and corms of Solomon’s lily were consumed (v. Kil 2:5; Shev. 5:2 and 4). Later recensions of the *De Materia Medica* include a reference to the *kolokasion*: “it is called alimon, some call it thymon, some, dracontium, and the Cyprians call it colocassion” [99]. This sentence was first added as a note in the margin of the 14th-century codex *Palatinus Graecus* of the Biblioteca Apostolica Vaticana, one of the most important testimonies of the text of Dioscorides [44], and is clearly the source of misidentification since Dioscorides’s description of *arum* does not correspond with taro. It is not certain that Dioscorides ever saw taro.

#### The Egyptian *aron* of Pliny

Pliny described the *arum* of Egypt as follows (*NH* 19,96) [41].

“Among the varieties of the bulb, too, there is the plant known in Egypt by the name of “*aron”*. In size it is very nearly as large as the squill, with a leaf like that of lapathum, and a straight stalk a couple of cubits in length, and the thickness of a walking-stick: the root of it is of a milder nature, so much so, indeed, as to admit of being eaten raw”.

Pliny’s *lapathum* is generally interpreted as a *Rumex* sp. [100], which only in case of *Rumex alpinus* L. has leaves that could be thought of as resembling those of taro. In terms of size, the corm of taro is comparable to the root of squill, *Drimia maritima* (L.) Stearn [97]. Although it is not generally regarded as being edible in the raw state, cultivated taro is likely to have less acridity than the wild aroids native to Egypt.

For Pliny, there were two different types of *arum*: a feminine one that was preferred for cooking and a masculine that was harder and more time-consuming to cook, and used to cure chest problems if dried and sprinkled in a drink (*NH* 24,143) [43]. During our fieldwork in Cyprus [2], the same gendered distinction was still made with regard to different forms of taro. Plants that were in the flowering phase at harvest were regarded as ‘male’, and produced harder corms, while those that were not flowering were regarded as ‘female’, and produced softer corms. The difference was not recognized by reference to flowering, but to the cross-section of cut petioles seen on corms sold in markets. An extra circle marked the ‘male’ plant, but anatomically, this marks the peduncle of an immature inflorescence that is not seen by the buyer.

#### The two *aron* types of Galen

In *On the Properties of Foodstuff*, Galen (Fig 2) described two types of *aron*: one used primarily for medicinal purposes and found in Greece, and one cultivated in Cyrene (Libya) as a nutritious food that was exported to Italy (Gal. *Al*. *Fac*. 2,65,1) [49].

*“On Arum*. The root of this plant is eaten much the same as that of the turnip, but in certain regions it grows somewhat more bitter; so that it is very like the root of the dracunculus [translated as edder-wort]. In cooking, one should pour off its first water and add more hot water, as was described in the case of cabbage and lentils. But in Cyrene the plant is the reverse of what it is in our country. For in those parts the arum has very little pharmacological activity and very little bitterness, so that it is more useful than turnips. Because of this they also export the root to Italy, on the grounds that it can keep for a very long time without rotting or sprouting. It is clear that this sort is better as nutriment, but if one wants to cough up any of the thick, viscid, fluids that accumulate in the chest and lung, the more bitter and more pharmacologically active root is better. When boiled in water, it is eaten with mustard or with oil, vinegar fish sauce, and of course with other mashed dishes, especially those prepared with cheese. But it is plain that the humor distributed from it to the liver and the body as a whole, from which animals are nourished, is somehow thicker, as was mentioned in the case of turnips. This is especially the case when the roots, like those from Cyrene, have no pharmacological activity. With us in Asia, many arums are more bitter and have medicinal property.”

Here, the medicinal plant described first is likely to be *Arum* or another wild aroid, while the Libyan plant is presumably taro, judging by the cooking method described, its mashability (a notable quality of boiled taro), and palatability (less bitter or acrid than other arums). Taro is still found growing in the Wadi Darnah [101], the only permanent river in Libya, which is located near the ancient Greek city of Cyrene, a major commercial hub in Greco-Roman times, near modern Shahhat. If in the 2nd century AD taro was indeed exported to Italy, this may explain the appearance of recipes for *colocasia* in Apicius.

#### *Arum* and *colocasia* in the Italian Renaissance botanical school

After Galen, scholars of the Mediterranean zone, whether Greek, Arabic or Latin, did not associate the name *arum* with taro and it took centuries before the edible *Cyrenaic arum* was finally identified with *colocasia*. During the Italian Renaissance, taro regained popularity among members of the botanical school led by the Professor of Medicinal Simples Luca Ghini at the Universities of Bologna (1534–1544) and Pisa (1544–1555) [102]. Ghini’s botanical school attracted scholars from across Europe after the Classical botanical treatises became more widely available in print. His students engaged in collecting, analyzing and classifying plants, with some of them travelling to faraway countries. Luigi Anguillara [56], one of Ghini’s students and subsequently prefect of the botanical garden in Padua (1545–1561), reported a conversation held in Cyprus between Sir Giovanni Battista Casanova and a Greek man who told Casanova that Cypriots knew taro as *colocasia*, and that the name was a very old term in their language. To further convince Casanova, the man showed him a Greek book of plants in which this name was present. Unfortunately, the book was not identified in Anguillara’s report.

After the botanical community in Italy learned about this encounter, scholars began to discuss different meanings of the name *colocasia* [103–105]. None of the Renaissance authors seems to have seen the *colocasia* of the ancient scholars (*N*. *nucifera*); living plants of the sacred lotus were probably rare in Egypt and Europe. Moreover, Silvatico’s *Pandette* [30] had not reached the wider scholarly audience, creating a discontinuity and confusion in botanical knowledge concerning aroids and lotus.

**Fig 3:**
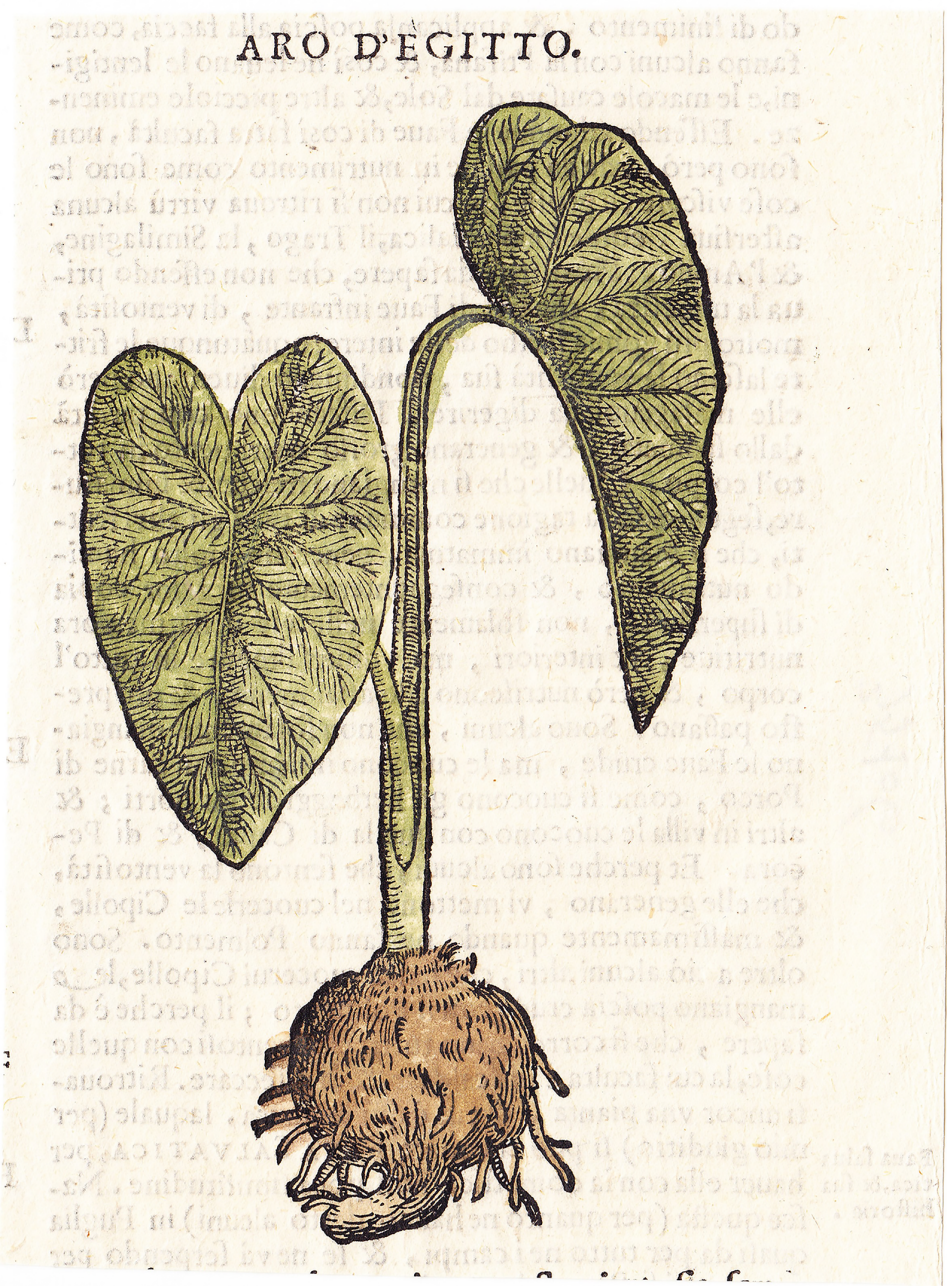
*Aro d’Egitto* in Mattioli 1580 ca. Commentarii. Drawing of taro made by Augier de Busbecq for the 1580 ca. edition of the Mattioli’s Commentarii [27].

Pietro Andrea Mattioli, who was well acquainted with the *Materia Medica* of Dioscorides [45], knew about the story that Anguillara reported. In the 1558 edition of the *MM*, under the chapter on the *Egyptian bean*, Mattioli wrote that he had seen this plant (taro) for the first time in Trieste in 1538 among other rare plants brought by Odoardo Polacco from one of his journeys in Syria and Egypt [103]. Mattioli examined the inconsistency of Classical authors’ descriptions and the plant that he had received. He concluded that those people who thought that this type of arum brought from Egypt (*Arum Aegyptium*) was the *Egyptian bean* (*N*. *nucifera*) were indeed wrong. In the 1565 edition of the *Discorsi* [32] he included a sketch of the plant named *Arum Aegyptium* (*Arum of Egypt*) that had been given by his friend Augier de Busbecq and that he used in the following editions. Despite the absence of inflorescence, the plant provided strongly resembles taro and is very different from lotus (Fig 3).

**Fig 4:**
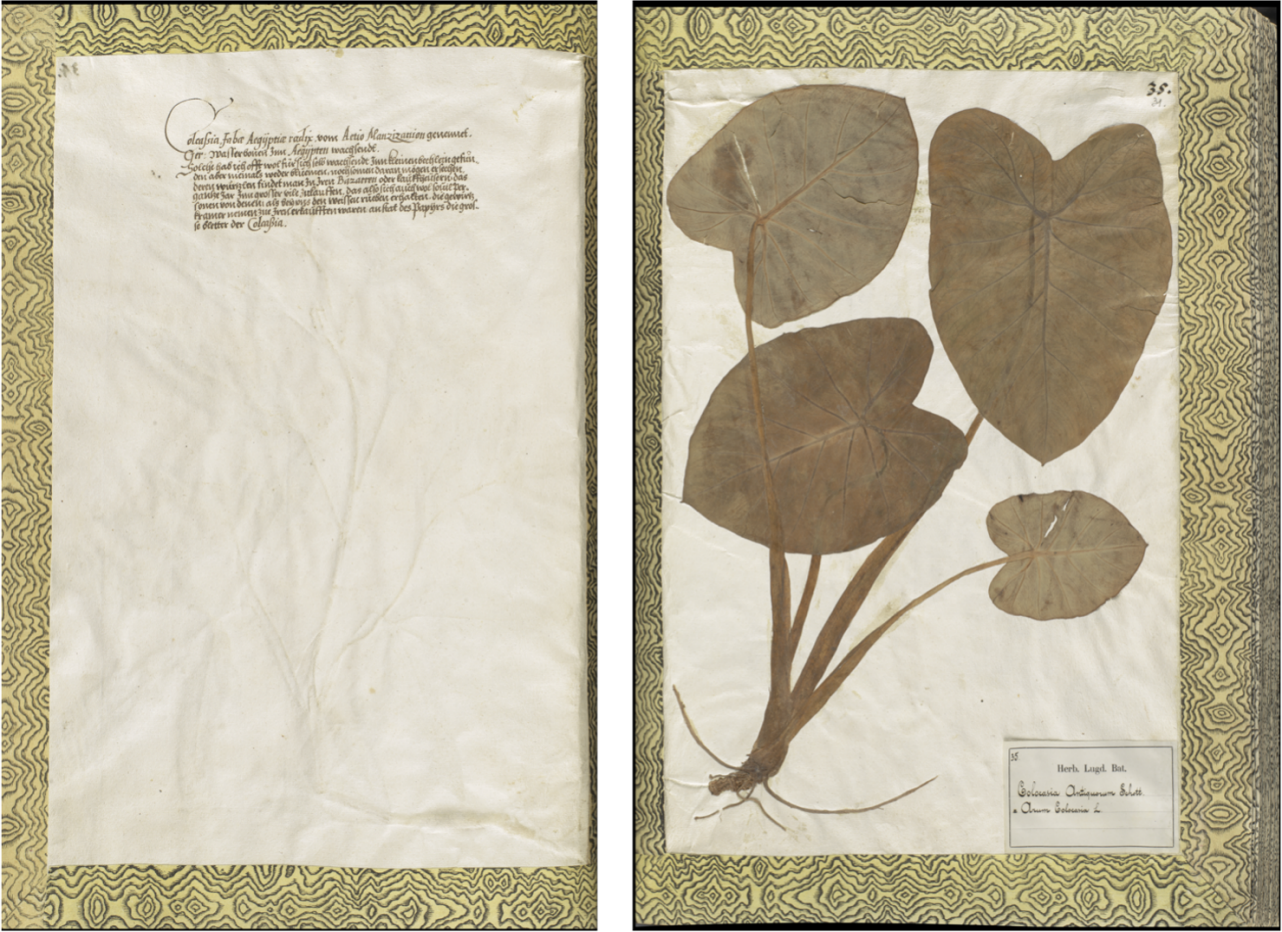
Colocasia in Rauwolf’s IVth herbarium. (A) Annotated text and (B) dried sample of *C*. *esculenta* collected by Rauwolf in Lebanon during his journey in the Middle East. Pictures by Naturalis Biodiversity Center.

In the following years, Renaissance botanists continued debating and developing their own ideas about the “real *colocasia* (Dioscorides’ *Egyptian bean*) and the common (taro) *colocasia*” [105], and included *Arum Aegyptium* in their observations [106–107], books [57, 59–60, 108, S11 Text] and herbaria [108]. While travelling in Lebanon and through the Ottoman Empire, the German botanist Leonhard Rauwolf [58] also collected taro, which he included in his herbarium with the name of Colocasia, the *Egyptian bean* (*Faba Aegyptia*) (Fig 4 A Fig 4 B)[S12 Text].

Rauwolf mentioned *colocasia* also in his travel accounts [59]. While in Nineveh (near Mosul, modern Iraq), he recalled the history of the Assyrian capital of Mesopotamia and commenting on its destruction by the Turko-Mongol emperor Timur in the 14th century, wrote that the city was left in such a devastating status that only *Beans* and *Colocasia* could grow there. We interpret *Colocasia* as taro, since Rauwolf had previously collected it in Lebanon (Fig 4B). The meaning of ‘Beans’ is less clear, but probably refers to the old name of *Egyptian bean* for sacred lotus, which Rauwolf gave to taro in his herbarium. His passage may imply that after the destruction of the city and its irrigated gardens, only crops of the riverside, taro and lotus, could continue to grow.

A century later, Paolo Boccone, a Sicilian botanist, wrote that people living in Mililli (current Melilli, Sicily), used to eat taro, known among them as *Culcasi* [see S8 Text], in times when wheat and bread were overpriced [61]. This indicates that taro was considered a substitute for wheat and served as a secondary, reserve crop, a role also noted in modern Cyprus [2].

## Discussion

The textual records, particularly those in Arabic, strongly suggest that by the 8th to late 12th centuries, taro was widespread in the Mediterranean as a food, medicinal and ornamental plant, and identified with various cognates of the names *qolqas* or *colocasia*. Early literature from the eastern Mediterranean suggests that taro reached the region during the late pre-Christian era, and was first associated with a name (*arum*) that was likely borrowed from the name for related wild plants already present in the region. Records from this era are sparse and do not suggest that the crop was common. The Hebrew evidence indicates that taro was present in Palestine from at least the 3rd century AD, with a new name (*karkas*) of unknown origin. There may have been a further linguistic shift from *karkas* to *qolqas* (Arabic for taro) as taro became more widespread in late Antiquity, while the sacred lotus became less common. The lotus eventually disappeared, possibly due to cultural changes in food preferences, and changes in land use and water control [19]. After the 3rd century AD (Fig 2), taro appears to be more widespread and identified by multiple names in different languages in the polyglot environment of the eastern Mediterranean. Widespread adoption of the Arabic name *qolqas*, from the Levant to Spain, indicates that the crop spread further with the expansion of Islamic agricultural systems [109].

The old suggestion, first articulated by de Candolle [21], that *kolokasia* is a term of Indic origin (Sanskrit *kāla-kacu*; taro) remains problematic on linguistic and semantic grounds [110]. The *kāla*- prefix in the Sanskrit word for taro is very rarely used in ancient texts, and can only mean 'black' or 'dark purple’ as it does today in the Assamese name for *kala-kochu*, a taro cultivar with dark petioles. Taro is more typically called *kacu* or *kacvī* in Sanskrit, and this is the form that survives in other Indo-Aryan languages, also as *kaachu* [111–112]. As the earliest attestations of *kolokasia* in the Mediterranean were only applied to the sacred lotus (*Nelumbo nucifera*), it is unlikely that *kolokasia* is related to the Sanskrit *kāla-kacu*. While the base-word *kasia* (κάσια) is of unknown etymology, the prefix *kolo-* was presumably added somewhere in the region between South Asia and Greece. Kronasser [113], Prellwitz [114] and Brust [110] have suggested that the prefix was a means to describe size and simply meant ‘large’, while Carnoy [115] argues that the suffix *kolo*- derives from the Traco-Pelasgian *gulo- ‘ball’ (cf. Sanskrit *gola* ‘ball’ or cylindrical as an adjective). Both suggestions are consistent with the large, round shape of taro mother-corms, but could also refer to large or globular forms of the edible rhizome of the sacred lotus.

*Kolokasia* (or *kolokasion*) does not resemble any of the many Indian names listed for *N*. *nucifera* by Sood and Prakash [112]. Our favored interpretation is that *kolokasia*, which emanates from the polyglot linguistic environment of the Eastern Mediterranean, was used first to describe the rhizomes of the sacred lotus, and only became associated with taro from the 4th century AD onwards. We have not addressed here all previous suggestions for the etymology of the Mediterranean names for taro, including those outlined in the extensive discussion by Täckholm and Drar [64]. A more detailed survey and analysis of past and present names is needed across all the regions where taro has been cultivated in southern and western Asia, northern Africa, and the Mediterranean.

### Introduction of taro to the Mediterranean

Our review of the historical sources suggests that taro arrived in southwestern Asia and the Mediterranean region by the 5th century BC and perhaps earlier. Like rice, for which literary evidence suggests cultivation in Mesopotamia from the 12th century BC [116], taro could have been grown in the flooded plains of ancient Iraq before being taken westwards to the Mediterranean.

Such a chronology for the introduction of the crop to the Mediterranean region fits with what we know generally about the exchange of domesticated crops and cattle in the Classical period. Textual sources, together with a growing archeobotanical record, suggest that many new plants and animals were introduced to the Mediterranean and Europe during this period. Many were initially rare exotics. Cats and chickens, for example, were present in Europe during the Hellenistic period (323–31 BC), but did not become more common until Roman times [117–118]. Plant introductions to Europe increased dramatically in the Roman period, and included at least cultivated vegetables, herbs, fruits and species, many of which spread as far as Britain, France and Germany [119–122]. The arrival of so many new species to Europe reflects a period of intensive travel and expanding interconnected trade networks that ultimately linked up eastern and western Eurasia. It is in the context of these intensified connections between east and west that taro probably made its way from more southern and eastern parts of Eurasia to southwestern Asia and ultimately the Mediterranean and northern Africa.

### Multiple names, multiple introductions?

The multiplicity of names used for taro in the Mediterranean suggests the possibilities of multiple routes of introduction and introduction of more than one cultivar. These possibilities can be explored further by comparison with linguistic and botanical data in historical records from across Eurasia and Africa. Although the present diversity of taro in the Mediterranean appears low, this could be the result of a single cultivar (clone) becoming dominant over time. The multiple names recorded may reflect multiple introductions, not all of which have survived. Evidence for past diversity might also emerge from further discoveries of archaeological tissue specimens. Other forms of archaeobotanical evidence can also be investigated: ancient residues of taro starch and calcium oxalate raphides have been reported from sites in Oceania [123–124], and may eventually be found in Mediterranean sites as the methods of residue analysis become more widely applied.

The intensity of trade and biological exchange in the ancient world adds to the likelihood that taro was introduced to the Mediterranean region more than once. While the Neolithic spread of agriculture often involved a limited range of crop species, diversification through the import of new crops and new varieties increased from the Bronze Age onwards [125]. Roman era trade led to the substantial diversification of domesticated plant and animal breeds and cultivars in the Mediterranean region, a process that intensified further in Medieval times.

While our review suggests the possibility of more than one pathway for taro into the Mediterranean, the limited diversity of the crop in the region today might in fact reflect a single or main ancient introduction. The recent global study of taro diversity [5] has demonstrated that individual clones of taro (specific cultivars) can be distinguished and tracked across vast distances. Genetic characterization of cultivars present in the Mediterranean today, and comparison with cultivars across the global range of taro, will also help to clarify the possible role of the Mediterranean as both source and sink in the wider history of taro. The semantic shift in the use of an earlier name for sacred lotus to a later name for taro also suggests a complex history for taro that is linked to wider changes in crop diversity and land use across the region. The role of taro as both a food and medicine in the ancient world must also have been significant for its perceived value for production and trade, and its dispersal along the terrestrial and maritime routes that carried trade products. The sacred lotus, with a longer history in the region, and more bountiful record in writing and art, may have been more important than taro in both economic and cultural terms. Yet, paradoxically, it is taro that survives today as a food crop in Egypt, while canalization of the delta and control of the Nile has largely removed the seasonally flooded habitats in which lotus long thrived.

## Supporting information

S1 Appendix Lists of references used to draw the map of taro distribution in the Mediterranean (Fig 1). (DOCX)

S1 Text Diphilos of Siphnos and Nicander of Colophon. (DOCX)

S2 Text Virgil’s colocasia. (DOCX)

S3 Text Dioscorides on the Egyptian bean. (DOCX)

S4 Text Columella’s colocasia. (DOCX)

S5 Text Pliny’s colocasia. (DOCX)

S6 Text Martial. (DOCX)

S7 Text Galen and Athenaeus. (DOCX)

S8 Text Anonymous plaque. (DOCX)

S9 Text Matteo Silvatico *Liber Pandectarum Medicinae* Chapter 197. (DOCX)

S10 Text Roccabonella *Liber de simplicibus*. (DOCX)

S11 Text Andrea Cesalpino *Liber XIV*. (DOCX)

S12 Text Colocasia in Rauwolf’s IVth herbarium 1583. (DOCX)
