# Supplementary material for: Literary evidence for taro in the ancient Mediterranean: A chronology of names and uses in a multilingual world
Source: PLoS One. 2018 Jun 5;13(6):e0198333. doi: 10.1371/journal.pone.0198333 (PMC5988270; doi:10.1371/journal.pone.0198333)
Supplement: S1 Appendix — (DOCX) [file pone.0198333.s001.docx]

**S1 Appendix 1: Supporting information for**

**Literary evidence for taro in the ancient Mediterranean: a chronology of names and uses in a multilingual world**

Ilaria Maria Grimaldi, Sureshkumar Muthukumaran, Giulia Tozzi, Antonino Nastasi, Peter J. Matthews, Nicole Boivin, Tinde van Andel

Appendix 1. Lists of references used to draw the map of taro distribution in the Mediterranean (Fig 1).

| **COUNTRY** | **LOCALITY and/or REGION** | **REFERENCES** |
| --- | --- | --- |
| **Algeria** |  | [1-4] |
|  | Cap Rosa | [5] |
|  | Stream Bou Redine, between El Kala and Annaba; Stream Oued en Nahal near Cap Rosa; Streams on the Beni Salah mountains | [6] |
| **Anatolia** |  | [3] |
| **Azores** |  | [2-3, 7] |
| **Balkan** |  | [3] |
| **Canaries** |  | [3] |
| **Cyprus** |  | [8] |
|  |  | [3] |
| **Egypt** |  | [2] |
| **Eritrea** |  | [3, 9] |
| **Egypt/Arabia** |  | [10] |
| **Greece** | Rhodes, Crete | [11] |
|  | Crete | [3] |
|  | Zante, Crete | [12] |
| **Italy** | Milis (Sardinia); Sicily; Calabria | [13] |
|  | Rome (Lazio); Naples (Campania) | [14] |
|  | Sicily (river Molinello; San Cosimano) | [15] |
|  | Sardinia, Sicily, Calabria | [3, 16-19] |
| **Lebanon** | Beirut, Tyre | [20] |
| **Libya** |  | [3, 21-23] |
| **Malta** |  | [16-17, 19, 25] |
|  | Bahria, Imtahleb, San Martin, Boschetto | [24] |
| **Morocco** |  | [3] |
| **Palestine** |  | [3, 26-27] |
|  | Wadi-Kilt | [20] |
| **Portugal** | Caldas de Monchique | [28] |
|  | Algarve | [29-30] |
|  | Madeira | [3, 31] |
| **Spain** | Malaga Province (Churriana, Alhaurin, Alhaurinejo) | [28] |
|  | Balearic Islands | [32] |
|  |  | [3, 33] |
| **Syria** |  | [3, 20] |
| **Tunisia** |  | [34] |
| **Turkey** | Içel province (Anamur and Bozyazi) | [35] |
|  |  | [36] |
| **Jordan** |  | [20] |
| **Former Yugoslavia (Bosnia and Herzegovina, Croatia, Macedonia, Slovenia, Serbia, Montenegro)** |  | [7, 37] |

References :

[1] Meyer A. Don précieu au amis, traitant des qualités des végétau et des simples. Alger: P. Fontana ; 1881.

[2] Durand T, Schinz H. Conspectus floræ Africæ: ou Énumération des plantes d'Afrique Vol. 5. Bruxelles: Jardin botanique de l'état ; 1895.

[3] Täckholm V, Drar M. Flora of Egypt, Vol. 2. Cairo: Fouad I University Press; 1950.

[4] Quézel P, Santa S, Schotter O. Nouvelle flore de l'Algérie et des regions désertiques méridionales Vol. 1. Paris: Ed. du Centre National de la Recherche Scientifique CNRS ; 1962.

[5] Battandier JA, Trabut L. Flore de l'Algérie contenant ka description de toutes les plantes signalées jusqu’à ce jour comme spontanées en Algérie et catalogue des plantes d'Algérie. Monocotylédones. Alger: Jourdan ; 1895.

[6] Maire R. 1957. Flore de l'Afrique du Nord (Maroc, Algérie, Tunisie, Tripolitaine, Cyrénaïque et Sahara) Vol. 4. Paris: P. Lechevalier ; 1957.

[7] Tutin TG. (ed.) (1980). Consolidated index to flora Europaea Vol. 5 Cambridge: University Press; 1980.

[8] Meikle RD. Flora of Cyprus, 2 vol. Kew Royal Botanic Gardens: Bentham-Moxon Trust; 1977-1985.

[9] Engels JM, Hawkes JG, Worede M. (editors.). Plant genetic resources of Ethiopia. Cambridge: Cambridge University Press; 1991.

[10] Hepper FN, Friis I. The plants of Pehr Forsskal's Flora Aegyptiaco-Arabica: collected on the Royal Danish expedition to Egypt and the Yemen 1761-63. Kew: Royal Botanic Gardens, Kew in association with the Botanical Museum, Copenhagen; 1994.

[11] Rechinger KH, Rechinger-Moser F. Flora Aegaea: Flora der Inseln und Halbinseln des Ägäischen Meeres. Vienna: Springer; 1943.

[12] Halacsy ED. Conspectus Florae Graecae. Lipsiae:  Engelmann; 1901-1904.

[13] Bertoloni A. Flora Italica: sistens plantas in Italia et in insulis circumstantib us sponte nascentes Vol. 10. Bologna: R. Masi; 1854.

[14] Parlatore F. Flora Italiana, ossia descrizione delle piante. Firenze: Le Monnier; 1857.

[15] Cavara F. Note floristiche e fitogeografiche di Sicilia. VII. La *Colocasia antiquorum* Schott nel territorio di Augusta. In: Bullettino della Società Botanica Italiana. Firenze: Stabilimento Pellas, 1905. pp. 137-145.

[16] Fiori A. Flora analitica d’Italia. Firenze: Tipographia di M. Ricci; 1908.

[17] Pignatti S. Flora d'Italia. Bologna: Edagricole; 1982.

[18] Matthews PJ. Written Records of Taro in the Eastern Mediterranean. In: Fusun Ertug Z, editor. Proceedings of the Fourth International Congress of Ethnobotany (ICEB 2005), Istanbul-Turkey, 21–26 August, 2005. Istanbul: Yayinlari; 2006. pp. 419-426.

[19] Fiori A. Nuova Flora Analitica d’Italia contenente la descrizione delle piante vascolari indigene inselvatichite e largamente coltivate in Italia Vol.1. Firenze: M. Ricci; 1923-1925.

[20] Post GE. Flora of Syria, Palestine and Sinai. Flora of Syria, Palestine and Sinai. 2nd edition). Beirut: Syrian Protestant College; 1933.

[21] Ringenbach J-C. Cyrenaica: The flowing wadis of the Jabal al Ackdar. 2014. Available at: http://jcringenbach.free.fr/website/habitats/cyrenaica_wadis.htm (Accessed 21 Sep 2013);

[22] El Gadi A. Flora of Libya: 41. Araceae. Tripoli: Al Faateh University; 1977.

[23] Keith HG. A preliminary check list of Libyan flora. Government of the Libyan Arab Republic, Ministry of Agriculture and Agrarian Reform; 1965.

[24] Borg J. Descriptive Flora of the Maltese Islands including the Ferns and Flowering Plants. Malta: Government Printing Office; 1927.

[25] Haslam SM, Sell PD, Wolseley PA. A flora of the Maltese Islands. Msida, Malta: Malta University Press; 1977.

[26] Dinsmore JE, Dalman G, Steuernagel DC. Die Pflanzen Palästinas: auf Grund eigener Sammlung und der Flora Posts und Boissiers verzeichnet. Leipzig: Hinrichs; Jerusalem: Vester; 1911.

[27] Oppenheimer HR. Florula transiordanica: révision critique des plantes récoltées et partiellement déterminées par Aaron Aaronsohn au cours de ses expéditions (1904–1908) en Transjordanie et dans le Wâdi el-‘Araba. Genève: Jent ; 1931.

[28] Willkomm M. Prodromus florae hispanicae: seu synopsis methodica omnium plantarum in Hispania sponte nascentium vel frequentius cultarum quae innotuerunt. Stuttgart: E. Schweizerbart ; 1861-1880.

[29] Coutinho AXP. Flora de Portugal. Paris: Aillaud, Alves ; 1913.

[30] Sampaio G.  Flora portuguesa. Pôrto: Imprensa Moderna; 1946.

[31] Press JR., Short MJ. Flora of Madeira. London: HMSO; 1994.

[32] Knoche H. Flora Balearica: étude phytogéographique sur les Iles Baléares. Montpelier : Roumégous et Déhan ; 1921-1923.

[33] Castroviejo S. Flora ibérica: plantas vasculares de la Península Ibérica e Islas Baleares. Madrid: Real Jardín Botánico, C.S.I.C ; 1996.

[34] Bonnet E, Barratte G. Catalogue raisonné des plantes vasculaires de la Tunisie. Paris: Inprimerie Nationale; 1896.

[35] Şen M, Akgül A, Özcan M. 2001. Physical and Chemical Characteristics of Taro (*Colocasia esculenta* (L.) Schott) corms and processing to chips and puree. Turk J Agric For. 2001; 25: 427-432.

[36] Thonner F. The flowering plants of Africa: an analytical key to the genera of African phanerogams. London: Dulau; 1915.
