# Supplementary material for: Literary evidence for taro in the ancient Mediterranean: A chronology of names and uses in a multilingual world
Source: PLoS One. 2018 Jun 5;13(6):e0198333. doi: 10.1371/journal.pone.0198333 (PMC5988270; doi:10.1371/journal.pone.0198333)
Supplement: S1 Text — (DOCX) [file pone.0198333.s002.docx]

**S1 Text: Supporting information for**

**Literary evidence for taro in the ancient Mediterranean: a chronology of names and uses in a multilingual world**

Ilaria Maria Grimaldi, Sureshkumar Muthukumaran, Giulia Tozzi, Antonino Nastasi, Peter J. Matthews, Nicole Boivin, Tinde van Andel

**Diphilos of Siphnos and Nicander of Colophon**

The earliest known uses of the term *kolokasia* appear in fragments of the works of Diphilos of Siphnos (early 3rd century BC), a physician at the court of the Macedonian king, Lysimachos, and those of Nicander of Colophon (2nd century BC), a poet-physician associated with the late Attalid court in Pergamon, Turkey. Their comments on *kolokasia* are preserved by the Roman gastronome Athenaeus of Naucratis (2nd century AD) in his text *The Banquet of the Learned* (*Deipnosophistae*) [1-2]. Diphilus of Siphnos refers to *kolokasion* (κολοκάσιον) as the root of the *Egyptian bean* (*Nelumbo nucifera*). He describes the food as tasty and nutritious, though hard to digest, and the beans as a laxative or a cause of flatulence (Athen. 3,72d–73a). Nicander describes the serving of the boiled tubers of *Egyptian bean* at banquets, and Athenaeus avows that Nicander means here what the Alexandrians (of Athenaeus’ time presumably, i.e. 2nd century AD) refer to as *kolokasion* (Athen. 3,72b). The cult of Athena Kolokasia at Sicyon (Corinth) is also mentioned by Athenaeus (Athen. 3,72b). As the lotus (*N. nucifera*) already bore associations with the sacred in Greek literature, this cult name is generally thought to refer to lotus, not taro.

[1] Yonge CD. The Deipnosophists: or, Banquet of the learned, of Athenæus. Vol. 1. London: HG Bohn; 1854.

[2] Griffiths M. The Lotus Quest: In Search of the Sacred Flower. New York: St. Martin's Press; 2010.
