# Supplementary material for: Literary evidence for taro in the ancient Mediterranean: A chronology of names and uses in a multilingual world
Source: PLoS One. 2018 Jun 5;13(6):e0198333. doi: 10.1371/journal.pone.0198333 (PMC5988270; doi:10.1371/journal.pone.0198333)
Supplement: S2 Text — (DOCX) [file pone.0198333.s003.docx]

**S2 Text: Supporting information for**

**Literary evidence for taro in the ancient Mediterranean: a chronology of names and uses in a multilingual world**

Ilaria Maria Grimaldi, Sureshkumar Muthukumaran, Giulia Tozzi, Antonino Nastasi, Peter J. Matthews, Nicole Boivin, Tinde van Andel

**Virgil’s colocasia**

In the *Eclogues* of Virgil (1st century BC), a mysterious millenarian child is lavished with beautiful plants as gifts, including *colocasia* (Virgil, *Ecl*. 4,18–20) [1]:

“But newborn boy, for you, these presents shall pour forth,

Cyclamen first, with ivy spread all over earth,

then colocasia mixed with acanthus smiling.” [2]

Martyn [3] sought to identify Virgil’s reference to *colocasia* as taro, although this appears unlikely on contextual and historical grounds. Fée [4] expanded on Martyn’s interpretation with some reservation, by noting that the original text contained words that did not suggest great splendor, but instead talked about first (*prima*), small presents (*munuscula*) produced without cultivation (*nullo culto*). It is the choice of words in Virgil that persuaded Fée to lean more towards the interpretation of *colocasia* as taro. If Virgil indeed refers to taro, it is unlikely that his *colocasia* refers to the (not particularly beautiful) roots, but to the flowers or whole plant, which is commonly considered beautiful today as it is an ornamental). The flowers of taro are less obvious and attractive than those of lotus, and as a fully aquatic plant, lotus may have appeared uncultivated to Virgil. It seems more likely that Virgil used the term *colocasia* to refer to lotus, the interpretation favored by most authors.

[1] Conington J. The poems of Virgil. London: Longmans, Green and Co; 1884.

[2] Krisak L. Virgil's Eclogues. Philadelphia: University of Pennsylvania Press; 2010.

[3] Martyn J. Publii Virgilii Maronis Bucolicorum eclogae decem: The Bucolicks of Virgil, Vol. 1. London: R. Reily. 1749; pp. 170–173.

[4] Fée ALA. Sur les Lotos des anciens: extrait de la Flore de Virgile. Lemaire. s.l. : s.n., 24 ; 1822.
