# Supplementary material for: Literary evidence for taro in the ancient Mediterranean: A chronology of names and uses in a multilingual world
Source: PLoS One. 2018 Jun 5;13(6):e0198333. doi: 10.1371/journal.pone.0198333 (PMC5988270; doi:10.1371/journal.pone.0198333)
Supplement: S3 Text — (DOCX) [file pone.0198333.s004.docx]

**S3 Text: Supporting information for**

**Literary evidence for taro in the ancient Mediterranean: a chronology of names and uses in a multilingual world**

Ilaria Maria Grimaldi, Sureshkumar Muthukumaran, Giulia Tozzi, Antonino Nastasi, Peter J. Matthews, Nicole Boivin, Tinde van Andel

**Dioscorides on the Egyptian bean**

In the great pharmacological compendium of Dioscorides, the *Materia Medica* (*MM*) (1st century AD), *colocasion* continued to be used as a term for the root (tuber) of the *Egyptian bean* (*Nelumbo nucifera*): (Dioscorides. *MM* 2,106)

‘The Egyptian bean, which some call *pontica*, grows abundantly in Egypt and it is found both in Asia and in Cilicia, in the marshes. It has a large umbellate leaf, a stalk that is a cubit long and thick as a finger, and a rose-coloured flower which is twice as large as a poppy and which, after it has finished blooming, bears a little sack that resembles a wasp’s nest. It contains the bean, which projects slightly beyond the cover, as if it were a bubble. It is called *ciborion* or *cibotion* because it is planted in moist lumps of earth and left this way in the water. The root is below, thicker than a reed. The root, which is called *colocasion*, is eaten both boiled and raw. This bean is eaten even green, but after it has been dried, it becomes black and it is larger than the Greek bean. It has astringent and wholesome properties. Their meal is suitable for people with colic and dysentery when sprinkled on their drinks instead of barley meal and it is also given as porridge...’ [1]

Everything in this account is consistent with *colocasion* referring to the root of *N. nucifera*, as the root (corm) of taro is never eaten raw because of its acrid properties [2].

[1] Beck LY. Pedanius Dioscorides De materia medica. Hildesheim, Germany: Olms-Weidmann; 2011.

[2] Matthews PJ. An introduction to the history of taro as a food. In V. Ramanatha Rao, PJ. Matthews, PB. Eyzaguirre and D. Hunter, editors. The Global Diversity of Taro, 6. 2010; pp.6-28.
