# Supplementary material for: Literary evidence for taro in the ancient Mediterranean: A chronology of names and uses in a multilingual world
Source: PLoS One. 2018 Jun 5;13(6):e0198333. doi: 10.1371/journal.pone.0198333 (PMC5988270; doi:10.1371/journal.pone.0198333)
Supplement: S4 Text — (DOCX) [file pone.0198333.s005.docx]

**S4 Text: Supporting information for**

**Literary evidence for taro in the ancient Mediterranean: a chronology of names and uses in a multilingual world**

Ilaria Maria Grimaldi, Sureshkumar Muthukumaran, Giulia Tozzi, Antonino Nastasi, Peter J. Matthews, Nicole Boivin, Tinde van Andel

**Columella’s colocasia**

In *De Re Rustica* (8,15,4), Columella (60s-70s AD), suggests planting *colocasia* in the middle part of decorative ponds “together with other green stuff which generally grows in the water and provides shade for the haunts of the waterfowl” [1]. Griffiths [2] interprets Columella as a reference to the sacred lotus, noting that images of lotus as a centerpiece of courtyard pools can be found among the buried mosaics and wall paintings of Pompei. However, in the courtyard gardens of Italy and Spain**,** there is a modern practice of planting taro on raised areas in the middle of ponds, often with waterlilies or lotus in the surrounding water. The reference to *colocasia* in Columella could therefore refer to either taro or lotus.

[1] Forster ES, Heffner E. Lucius Junius Moderatus Columella. On Agriculture. London: Heinemann; Cambridge, Mass.: Harvard University Press; 1968.

[2] Griffiths M. The Lotus Quest: In Search of the Sacred Flower. St. Martins; 2010.
