# Supplementary material for: Literary evidence for taro in the ancient Mediterranean: A chronology of names and uses in a multilingual world
Source: PLoS One. 2018 Jun 5;13(6):e0198333. doi: 10.1371/journal.pone.0198333 (PMC5988270; doi:10.1371/journal.pone.0198333)
Supplement: S5 Text — (DOCX) [file pone.0198333.s006.docx]

**S5 Text: Supporting information for**

**Literary evidence for taro in the ancient Mediterranean: a chronology of names and uses in a multilingual world**

Ilaria Maria Grimaldi, Sureshkumar Muthukumaran, Giulia Tozzi, Antonino Nastasi, Peter J. Matthews, Nicole Boivin, Tinde van Andel

**Pliny’s colocasia**

In the *Natural History* (*NH*), in a section on Egyptian plants, and drawing from the work of Theophrastus, Pliny the Elder (1st century AD) stated (Plin. *NH* 21,87):

“There follow the plants that grow wild. Most peoples use these for food, especially the people of Egypt, a land very fruitful of crops, yet about the only one that could manage without them, so great an abundance of food does it get from [wild] plants…

In Egypt the most famous plant of this kind is the *colocasia*, called by some *cyamos* [bean, i.e. *Nelumbo nucifera*]; they gather it out of the Nile. The stalk of the stem when boiled and chewed breaks up into spidery threads, but the stem itself is handsome, jutting out from leaves which, even when compared with those of trees, are very broad, similar to the leaves called *personata* which are found in Italian rivers. So much do the people of the Nile appreciate the bounty of their river that they plait *colocasia* leaves into vessels of various shapes, which they consider make attractive goblets. The *colocasia* is now grown in Italy”. [1]

Past interpreters have attempted to identify different parts of Pliny’s description with *Nelumbo nucifera* and *Colocasia esculenta* [2], but in fact the description as a whole is consistent with just one plant, the Egyptian bean (*N. nucifera*) and its known multiple uses. An additional clue is provided by the context, as Pliny is describing a plant that is abundant and wild in the Nile. This can only refer to a fully aquatic plant such as *N. nucifera*, which was certainly famous in the ancient world [3]. The fibres of lotus stems were also described by Martial.

[1] Jones WHS. Pliny Natural History in ten volumes. Vol. VI Libri XX-XXIII. London: William Heinemann LTD; Cambridge, Ma: Harvard University Press; 1969.

[2] Bostock J, Riley HT. Pliny the Elder. The Natural History Vol. IV. London: Henry G. Bohn; 1856.

[3] Griffiths M. The Lotus Quest: In Search of the Sacred Flower. St. Martins; 2010.
