# Supplementary material for: Literary evidence for taro in the ancient Mediterranean: A chronology of names and uses in a multilingual world
Source: PLoS One. 2018 Jun 5;13(6):e0198333. doi: 10.1371/journal.pone.0198333 (PMC5988270; doi:10.1371/journal.pone.0198333)
Supplement: S6 Text — (DOCX) [file pone.0198333.s007.docx]

**S6 Text: Supporting information for**

**Literary evidence for taro in the ancient Mediterranean: a chronology of names and uses in a multilingual world**

Ilaria Maria Grimaldi, Sureshkumar Muthukumaran, Giulia Tozzi, Antonino Nastasi, Peter J. Matthews, Nicole Boivin, Tinde van Andel

**Martial**

The Roman poet Martial (late 1st century AD) also mentions *colocasia*, and in his *Epigrammata* describes the plant as having a slender stalk (8,33,13), and as a vegetable with ‘strong threads’ and stubborn or clinging fibres (13,57):

“Colocasia

You will laugh at the vegetable from the Nile and its clinging fibres,

when you pull at its naughty threads with mouth and fingers.”

(Martial, *Epigrammata* 13,57; translation by IMG).

While Ker [1] translated *colocasia* in this passage as the *Egyptian bean* (*Nelumbo nucifera*), Shackleton Bailey [2] identified this as taro, noting that there is confusion with *N. nucifera*. However, both the corms and leaf stems of taro are soft when cooked, while the rhizomes of *N. nucifera* are stringy [3]. The petiole produces fibres that are spun into lamp wicks used during traditional Hindu rituals [4]. The fibres are also used to weave an expensive fabric worn by Buddhist monks in Myanmar and employed by some modern Italian suit makers [5]. Kintaert [6] notes Sanskrit references to clothes made from the fibres of lotus rhizomes, and body ornaments made from lotus stalk fibres.

[1] Ker WCA. Martial Epigrams. Volume II. London: William Heinemann; 1920.

[2] Shackleton Bailey DR. Martial Epigrams, Vol. III. Cambridge, Massachusetts; London: Harvard University Press; 1993.

[3] Li Z, Liu X, Gituru RW, Juntawong N, Zhou M, Chen L. 2010. Genetic diversity and classification of *Nelumbo* germplasm of different origins by RAPD and ISSR analysis. Scientia Horticulturae. 2010; 125(4): pp. 724-732.

[4] Arundhati P. Royal life in Mānasôllāsa. Delhi: Sundeep Prakashan, 29; 1994.

[5] Brinkley C. New Luxury Frontier: A $5,600 Lotus Jacket. The Wall Street Journal, Nov. 3, 2010. http://on.wsj.com/1w67imV (30 Mar 2015); 2010.

[5] Kintaert T. On the Cultural Significance of the Leaf of the Indian Lotus: Introduction and Uses. From Turfan to Ajanta, 2010; 481-512.
