# Supplementary material for: Literary evidence for taro in the ancient Mediterranean: A chronology of names and uses in a multilingual world
Source: PLoS One. 2018 Jun 5;13(6):e0198333. doi: 10.1371/journal.pone.0198333 (PMC5988270; doi:10.1371/journal.pone.0198333)
Supplement: S7 Text — (DOCX) [file pone.0198333.s008.docx]

**S7 Text: Supporting information for**

**Literary evidence for taro in the ancient Mediterranean: a chronology of names and uses in a multilingual world**

Ilaria Maria Grimaldi, Sureshkumar Muthukumaran, Giulia Tozzi, Antonino Nastasi, Peter J. Matthews, Nicole Boivin, Tinde van Andel

**Galen and Athenaeus**

The Greek physician Galen **(**2nd c. AD) lived in Italy and Turkey, and had a strong interest in useful plants, their accurate identification, and the qualities of their products [1].

In a chapter on wild plants in his *On the Properties of Foodstuff*, Galen stated: ‘Not only do we eat the seeds and fruits of plants, but also the plants themselves, often whole, but often only the roots, branches or young shoots, according as there is a pressing need for each…’ These are plants that give ‘little nutriment’ and are ‘unwholesome’, and include the ‘*kibôria* and *kolokasia*’ (*Al. fac.* 2,39,4). Earlier authors identified these as the fruit and root of the same plant, namely the Egyptian bean (*N. nucifera*). After dealing with the wild plants, Galen discussed garden plants, among which *kolokasia* is not mentioned. Apparently, Galen’s *kolokasia* does not refer to cultivated taro with its large and nutritious corms.

While recounting the observations of earlier authors regarding the *kolokasion* (root) of Egyptian bean (*N. nucifera*), Athenaeus (2nd century AD) noted that the name is similarly used in Alexandria, thus indicating that the usage remained current in the 2nd century.

[1] Powell O. Galen. On the Properties of Foodstuffs (De alimentorum facultatibus). Cambridge and New York: Cambridge University Press; 2003.
