# Supplementary material for: Literary evidence for taro in the ancient Mediterranean: A chronology of names and uses in a multilingual world
Source: PLoS One. 2018 Jun 5;13(6):e0198333. doi: 10.1371/journal.pone.0198333 (PMC5988270; doi:10.1371/journal.pone.0198333)
Supplement: S8 Text — (DOCX) [file pone.0198333.s009.docx]

**S8 Text: Supporting information for**

**Literary evidence for taro in the ancient Mediterranean: a chronology of names and uses in a multilingual world**

Ilaria Maria Grimaldi, Sureshkumar Muthukumaran, Giulia Tozzi, Antonino Nastasi, Peter J. Matthews, Nicole Boivin, Tinde van Andel

**Anonymous plaque**

The epithet *kolokasion* was found on a marble plaque dated to the 4th or 5th century AD, and dedicated to a Jewish man whose nickname was *kolokasion*: "Grave of Jeremias Kolokasios. Iose and Theodotous (who) (made this) for their son” [1]. Although the circumstances of its original acquisition are unknown, the plaque is supposed to come from the Syro-Palestinian coast. The name engraved on the plaque was interpreted by Schwartz [1] as a reference to the edible root of the Egyptian bean (*N. nucifera*). There is nothing, however, in the plaque itself to support any particular interpretation of the term *colocasia*. However, the use of a plant name to identify people has been reported for many languages. In Sicily, *Culcasi* and *Culcasio* are recorded from the 13th century as common surnames of Greek origin, associated with the plant *colocasia* [2]. In Cyprus, the Greek name for taro (*kolokasi*) was adopted as a formal surname (*Colocasides*) by several families when the British administration forced islanders to use the surname, first name system. According to one member of the *Colocasides* family, the name had been given to an ancestor because of his greedy appreciation of taro served at a wedding banquet (Matthews, unpublished field notes).

[1] Schwartz S. 1989. A Greek Inscription in the Library of the Annenberg Research Institute. The Jewish quarterly review. 1989; 80 (1–2): 87-91.

[2] Blunda Supposte origini dei cognomi di Sicilia. <http://www.trapaninostra.it/libri/Vito_Blunda/Supposte_origini_dei_cognomi_in_Sicilia/Supposte_origini_dei_cognomi_in_Sicilia.htm> (25 Jun 2014); 2014.
