# Supplementary material for: Literary evidence for taro in the ancient Mediterranean: A chronology of names and uses in a multilingual world
Source: PLoS One. 2018 Jun 5;13(6):e0198333. doi: 10.1371/journal.pone.0198333 (PMC5988270; doi:10.1371/journal.pone.0198333)
Supplement: S9 Text — (DOCX) [file pone.0198333.s010.docx]

**S9 Text: Supporting information for**

**Literary evidence for taro in the ancient Mediterranean: a chronology of names and uses in a multilingual world**

Ilaria Maria Grimaldi, Sureshkumar Muthukumaran, Giulia Tozzi, Antonino Nastasi, Peter J. Matthews, Nicole Boivin, Tinde van Andel

**Matteo Silvatico** *Liber Pandectarum Medicinae*

Chapter 197

*Culcasia, culcas* or *collocasia* in Greek, *hulcas* in Arabic, indeed *caso* in Latin. Pliny, in a chapter on *Culcasia*’s virtues: *Culcasia*, that somebody calls *cyamos*: the most famous in Egypt, they reap it from the Nile, with the stalk that once cooked, when is chewed it is thready, but it has a handsome thyrse that stands among the leaves that are very broad, even when they are compared with those of the trees, like those which we call in our region *Personata* (they are called *Lappago Maior* in accordance with Dioscorides) and so much are they pleased with the virtues of their Nile plant that they consider it very attractive to drink from the leaves of the *colocasia* intertwined in various shapes of vessels, etc.[In *Pandette*, Pliny’s text is quoted with some errors: the correct one is translated here.]This is the herb that Dioscorides calls *faba egyptiaca*: you will find the properties and the virtues of this plant in accordance with him under the term *faba egyptiaca*; and Avicenna calls *hulcas* this *faba egyptiaca*, about which he writes a chapter in the second book of the Canon [The Canon of Medicine]. Indeed *culcasia* is very well known in Egypt among the merchants who trade in Syria. And I have the plant itself in Salerno in my garden near a beautiful spring: *culcasia*, which in our common language is called *caso*. The leaves of this plant, full of water like vessels, delight the minds of the ill and the convalescent, and indeed excite the libido.

Serapion, in the Book of Aggregations [*Liber Aggregatus*], in the chapter on *Hulcas*, based on the authority of Galen: *Hulcas* i.e. *culcasia*. This plant has a very acrid and sharp taste and it is not very astringent. And its virtue is hot at the second degree and, once cooked, helps the stomach. And the same based on the authority of Aben Mesuay: *Culcasia* is hot and humid and increases the sperm and the root is cooked and has a good taste; it is good for the stomach and provokes appetite and urine. It is hot and humid and increases the sperm. This plant is very familiar to the Egyptians and springs up near the water, and it has large and big leaves and the root once cooked and eaten increases the body. Paulus in the chapter about *culcasia*: *Culcasia* is a plant known by everyone. It springs up near the water; its root once cooked and eaten is very useful to the stomach. Isaac in the *Particular Treatments* in the chapter about *culcasia*: *Culcasia* is a plant that springs up mostly in Egypt and it is slightly bitter and pungent whence it is hot and humid. The root of this plant, once stewed, gives off an acrid juice; and the viscosity that before was hidden becomes visible. And for this reason it produces strong and immature nourishment, but with its bitterness supports the stomach and constipates the belly. However, if they eat it moderately, it is good nourishment; and it is good for people who suffer from dysentery given its viscosity and bitterness.

Avicenna, Book II, chapter *Hulcas or culcasia*: It is a plant which bears similarity with the *alisnet* [a plant not yet identified]. It is hot and dry at the first degree; and it is salty and astringent; and its parts are not similar; and it gives little flatulence. Gargles are made with its same milk and salt; and in Egypt it is cooked with meat; and it is fried with oil; for them it is food; and mashed it releases yellow water and properly its seed and its juice, and so what debilitates is decreased; it causes urine and generates sperm and expels easily gall and water; drink from 1/3 to 2/3 of 1 lb of it.” (Translation by GT and AN)

Silvatico M. Liber pandectarum medicinæ (opus emēdatū per ... dominū Matheum moretū). Chapter 197 Culcasia. Translation by Giulia Tozzi e Antonino Anastasi following the text of the Venetian print-edition of 1488 (Matthaeus Silvaticus, Liber Pandectarum Medicinae, Venetia: Marinus Saracenus, f. 77; 1474). Bologna: I. Vurster; 1488.
