# Supplementary material for: Literary evidence for taro in the ancient Mediterranean: A chronology of names and uses in a multilingual world
Source: PLoS One. 2018 Jun 5;13(6):e0198333. doi: 10.1371/journal.pone.0198333 (PMC5988270; doi:10.1371/journal.pone.0198333)
Supplement: S10 Text — (DOCX) [file pone.0198333.s011.docx]

**S10 Text: Supporting information for**

**Literary evidence for taro in the ancient Mediterranean: a chronology of names and uses in a multilingual world**

Ilaria Maria Grimaldi, Sureshkumar Muthukumaran, Giulia Tozzi, Antonino Nastasi, Peter J. Matthews, Nicole Boivin, Tinde van Andel

**Roccabonella** *Liber de simplicibus*

The text [1] is written in Medieval Latin handwriting and the Latin text with an English translation is reported here:

*“De culcasia. Capitulum 114.*

*Herba picta in hac carta infra scriptis appellatur nominibus.*

| ***Graece*** | ***Latine*** | ***Arabice*** |
| --- | --- | --- |
| *Faba Egipciaca* | *Colocasia* | *Culcas* |
| *Faba Sira* |  | *Culcasia* |
| *Ciamon* |  | *Hulcas* |

*In medicinis utimur herba et suco eius et radice. Colligi potest tam herba et exprimi sucusquam radix, a mense Iunii usque ad finem Septembrem, quamquam herba melius de mense Iunii et radixmensem Septembrem, luna crescente. Servari potest per annum, quamquam recens melioris sit operationis. De ea tractat Serapio libro suo de Simplicibus, capitulo de hulcas, idest culcasia. Et Avicenna secundo canone, capitulo de culcasia. Et Simon Ianuensis et Mundinus in suis Sinonimis, capitis de culcas, et de hulcas, et de ciamon, et de colocasia, et de faba Egipciaca. Et Galenus librode alimentis.*

(Niccolò Roccabonella, *Liber de Simplicibus*, Marc. Lat. VI 59 (=2548), 114v)

Culcasia. Chapter 114.

The herb drawn in this page is called by the following names:

| **Greek** | **Latin** | **Arabic** |
| --- | --- | --- |
| Egyptian bean | *Colocasia* | *Culcas* |
| Syrian bean |  | *Culcasia* |
| *Ciamon* |  | *Hulcas* |

In medicine we use the herb and its juice and root. It is possible to gather both the herb, and to squeeze out the juice from it, and the root from the month of June until the end of September, although it is better to collect the herb from the month of June and the root in the month of September, during the waxing moon. It can be kept for one year, even though the fresh variety is preferable. Serapion treats it in his book “de Simplicibus”, in the chapter on *Hulcas*, i.e. *culcasia*. And Avicenna writes about it in the second book of the Canon, in the chapter on *Culcasia*. And Simon of Genoa and Mundinus in the *Synonyms* in chapters about *culcas*, *hulcas*, *cyamon*, *colocasia*, and the Egyptian bean. And it is mentioned by Galen too in his book “On the Properties of Foodstuffs”. (Translation made by Grimaldi I.M.)

[1] Roccabonella N. 1419 (1445-1448). *Liber De Simplicibus*, Venezia, Marc. Lat. VI 59 [=2548]).
