# Supplementary material for: Literary evidence for taro in the ancient Mediterranean: A chronology of names and uses in a multilingual world
Source: PLoS One. 2018 Jun 5;13(6):e0198333. doi: 10.1371/journal.pone.0198333 (PMC5988270; doi:10.1371/journal.pone.0198333)
Supplement: S11 Text — (DOCX) [file pone.0198333.s012.docx]

**S11 Text: Supporting information for**

**Literary evidence for taro in the ancient Mediterranean: a chronology of names and uses in a multilingual world**

Ilaria Maria Grimaldi, Sureshkumar Muthukumaran, Giulia Tozzi, Antonino Nastasi, Peter J. Matthews, Nicole Boivin, Tinde van Andel

**Andrea Cesalpino** *Liber XIV*

Standard font is used for Cesalpino’s own observation, italics mark passages derived from previous authors, and square brackets mark our comments:

*ARON MAGNUM*

Aron magnum, which is commonly called colocasia, sprouts in Sicily: with broad leaves, of a size (similar) to the personata [*Arctium lappa* L.], in the aspect to the Gighero [*Arum maculatum* or *A. italicum*], but with the lower corners of the leaves joined, so that a sort of concavity is formed in the leaf, with an extremely smooth surface and extremely lush, with variation in colors and resembling verdant and luxuriant waves. A thick pedicle the thickness of a walking stick, one cubit long and more, and from where a leaf that faces downwards hangs [one cubit equals the length of an average lower arm, from the elbow to the tip of middle finger]. The inhabitants [of Sicily, or perhaps generally the Italian Peninsula] call them ‘Pampina Paradisi’ [literally ‘Heaven’s fronds’]: they use the leaves to preserve fresh fruits and fresh cheese. The underground root is round and thick, like the one of the dracunculus maior, certainly bitter, but with an innocuous taste. This does not carry a stem, nor a fruit; it becomes lush during the entire summer, it loses its leaves during the winter, and it needs abundant water. Theophrastus calls it ‘aron’ and ‘edible aron’. He reports this information about it here and there. *The very broad leaves are scattered and they are concave and large like the ones of the Cucumeracea. There is no stem, nor a flower, the root is edible also the leaves once cooked in vinegar* [cf. Theophr. *HP* 7,13,1–2]*. It is agreeable to the taste, and good for fractures. The root is soft throughout, almost without skin, and it is fleshy. It has another root, thin and with tough fibres, such as to call into doubt the use of the term ‘root’ as also in cyperus*. *When the root is thriving with the leaves, in order to increase growth, the inhabitants used to cover it all around, bending the leaves themselves, so that the plant does not grow on their buds, but drags all the nutriment to its head* [cf. Theophr. *HP* 1,6,8–10] [a precise description of how cultivated taro is commonly managed towards the end of the growing period]. *Pliny, talking about aron, similarly considers this edible, and there is much controversy over its relationship to the dracontium* [*Dracunculus vulgaris* Schott]*. Some said that they are the same thing. Glaucias distinguished them from the place of growth, calling dracontium the wild aron. Others call the root aron, and the stem dracontium, which is completely different. The root of the arum is black, broad and round, and much bigger so that it fills the hand, much revered both as food and as medicament. It is said that cheese is best kept in the leaves of arum* [cf. Plin. *NH* 24,142–148]*. Among the different types of bulbs in Egypt, there is the one that they call arum, similar in size to the scyllae* [*Drimia maritima* (L.) Stearn]*, and in its leaves to the lapathum* [*Rumex acetosa* L.]*. The stem is straight, with the thickness of a walking stick of two cubits, and with a soft root, which is edible even eaten raw* [cf. Plin. *NH* 19,96]*.* But we have not seen this Egyptian type (of arum) yet. For this reason, it can be inferred that Dioscorides’s manuscripts are corrupted, because many traits that are characteristic of edible arum, are instead attributed to dracontium minor, like these: *cheese wrapped in the leaves is preserved from rotting; the root, either cooked or raw, is used as a vegetable to improve health; at banquets in the Balearic Islands, instead of cakes, they offer the root cooked with a lot of honey* [cf. Dioscor. *MM* 2,197]. And some traits that are characteristic of dracontium minor are attributed to aron, like the saffron-colored fruits. However, among other names, in Dioscorides it is said that Cypriots call it colocasion, a name still maintained among us, even though it is confused with the Egyptian bean that Pliny says is called colocasia (Translation by Tozzi G and Nastasi A.).

The name *colocasion* or *colocasia* that Cesalpino refers to above was introduced into later editions of Dioscorides by 16th c. interpreters. Cesalpino recognized that the name was originally used to indicate Egyptian bean, i.e. *Nelumbo nucifera*.

[1] Cesalpino A. De Plantis libri xvi. Florentiae: apud Georgium Marescottum; 1583.
