# Supplementary material for: Literary evidence for taro in the ancient Mediterranean: A chronology of names and uses in a multilingual world
Source: PLoS One. 2018 Jun 5;13(6):e0198333. doi: 10.1371/journal.pone.0198333 (PMC5988270; doi:10.1371/journal.pone.0198333)
Supplement: S12 Text — (DOCX) [file pone.0198333.s013.docx]

**S12 Text: Supporting information for**

**Literary evidence for taro in the ancient Mediterranean: a chronology of names and uses in a multilingual world**

Ilaria Maria Grimaldi, Sureshkumar Muthukumaran, Giulia Tozzi, Antonino Nastasi, Peter J. Matthews, Nicole Boivin, Tinde van Andel

**Colocasia in Rauwolf’s IVth herbarium 1583**

The text [1] is written in Medieval Latin handwriting and the Latin text with an English translation is reported here:

“Colcasia, Faba Aegijptiae radix, vom Aetio Manzizanion genennet. Ger. Wasserbonen in Aegÿpten wachsendt - Solche hab ich offt wol für sich selb wachsendt inn kleinen bechlein gefůnden aber niemals weder blüemen, noch somen daran mögen ersechen, deren wůrtzlen findet man In Iren Bazarren oder kauffheüsern das ganze Jar Inn grosser wile zukauffen, das also sich auch wol sovil personen von denen als beij uns den weissen rüeben erhalten. die gewirtz kramer nemen zue Iren erkaüfften waren an stat des Papijrs die grosse bletter der Colcaßia"

"Colcasia. Root of Egyptian bean, named Manzizanion by Aetio.

German: waterbeds growing in Egypt.

Such I have often found growing by itself in small streams, but I was never able to see flowers or seeds on it. Its roots are found in great quantities in their Bazars or shops throughout the year, that also so many people [buy this] like [they do] white turnip in our country. The plant vendors offer their goods for sale, instead of Papyrus, on the large leaves of Colocassia".

Translation made by Ghorbani A.
